# Targeting Caveolin-1 for enhanced rotator cuff repair: findings from single-cell RNA sequencing

**DOI:** 10.1038/s41420-025-02359-2

**Published:** 2025-03-05

**Authors:** Shanhong Fang, Songye Wu, Peng Chen

**Affiliations:** 1 Department of Orthopedic Surgery, The First Affiliated Hospital of Fujian Medical University, Fuzhou, PR China; 2 Department of Sports Medicine, National Regional Medical Center, Binhai Campus of the First Affiliated Hospital, Fujian Medical University, Fuzhou, PR China; 3 Fujian Orthopaedics Research Institute, Fuzhou, PR China; 4 Fujian Orthopedic Bone and Joint Disease and Sports Rehabilitation Clinical Medical Research Center, Fuzhou, PR China

**Keywords:** Diseases, Cell biology

## Abstract

Rotator cuff injury (RCI), a prevalent cause of shoulder pain and disability, often leads to significant functional impairments due to adipocyte infiltration into the damaged tissue. Caveolin-1 (Cav-1), a critical membrane protein, plays a significant role in adipocyte differentiation and lipid metabolism. This study utilized single-cell RNA sequencing (scRNA-seq) to investigate the heterogeneity of cell subpopulations in RCI tissues and assess the regulatory effects of Cav-1. The findings revealed that Cav-1 expression negatively correlates with adipogenic activity, and its modulation through exercise or targeted therapies can significantly reduce adipocyte infiltration and enhance tissue repair. Further, Cav-1 knockout and overexpression models demonstrated the protein’s impact on key genes involved in adipocyte differentiation and lipid metabolism, such as Scd1, fatty acid synthase (FASN), and peroxisome proliferator-activated receptor gamma (Pparg). Animal studies corroborated these results, showing that exercise intervention increased Cav-1 expression, decreased adipocyte infiltration, and promoted structural repair. These insights suggest that targeting Cav-1 could offer a novel therapeutic strategy for improving RCI outcomes.

## Introduction

Rotator cuff injury (RCI) is a common shoulder joint disease characterized by fat infiltration, which is one of its major pathological features [1]. Infiltration of adipocytes is a significant factor in hindered tissue repair and functional recovery due to RCI [2]. An abnormal increase of fat cells in RCI may lead to inflammation and cell apoptosis, further affecting the tissue repair process of the rotator cuff (RC) [3–5]. Therefore, investigating the molecular mechanisms of fat infiltration in RCI is important for improving the therapeutic outcomes of RCI patients [4, 5].

In recent years, single-cell RNA sequencing (scRNA-seq) has become a crucial technique for studying the molecular mechanisms of fat infiltration in RCI [6–8]. This technique enables in-depth analysis of the heterogeneity of different cell subpopulations in RCI tissues [9, 10]. Based on the results of molecular biology experiments, we discovered that Caveolin-1 (Cav-1) exhibits significant changes in expression level during fat infiltration in RCI patients and shows a close correlation with the expression of genes related to fat cell differentiation.

Cav-1, an important cell membrane protein, has been found to play a critical role in many cellular processes [11–13]. To study the role of Cav-1 in fat cell differentiation, we constructed 3T3-L1 cell models with Cav-1 knockout or overexpression using CRISPR/Cas9 gene editing technology or lentiviral transfection. Through these models, we demonstrated that Cav-1 significantly regulates fat cell differentiation and exhibits a certain correlation with gene expression related to lipid metabolism.

Exercise, as a non-surgical treatment method, is widely applied in RCI patients with proven effectiveness [14–16]. However, the molecular mechanisms of exercise on fat infiltration remain unclear [17]. To investigate the effects of exercise on fat infiltration in RCI patients further, we constructed an RCI animal model and used treadmill exercise or Cav-1 antibody treatment to evaluate their impact on fat infiltration and structural repair in the RCI area.

In summary, this study aims to investigate the molecular mechanisms of exercise-mediated regulation of Cav-1 expression in fat infiltration in RCI patients. The results suggest that exercise, as a non-surgical treatment method, has important scientific and clinical significance by reducing fat cell infiltration and promoting structural repair in the RCI area. This study provides new molecular biology evidence and potential therapeutic targets for the non-surgical treatment of RCI, offering new treatment strategies and directions for clinical practice. We hope that this research can provide new ideas and methods for the rehabilitation of RCI patients.

## Results

### Role of Cav-1 and lipidogenesis in RCI development

**Fig. 1:**
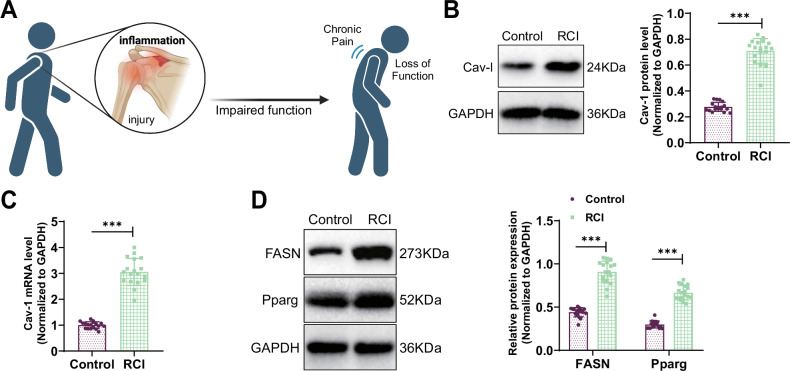
Correlation analysis of Cav-1 with clinical significance. **A** Schematic illustration of the development process of limb dysfunction and chronic pain after shoulder muscle injury. **B** Western blot analysis of Cav-1 protein expression in shoulder samples collected from Control (*n* = 18) and RCIl (*n* = 18) sources. **C** RT-qPCR analysis of Cav-1 mRNA expression in samples from Control and RCI sources. **D** Western blot analysis of FASN and Pparg protein expression in samples from Control and RCI sources; *** indicates *P* < 0.001.

In our preliminary research [18], we found a close association between Cav-1 and RCI through in vitro experiments. Building upon this discovery, we examined the shoulder RC tendon tissues of both the Control and RCI group (Fig. 1A). The results indicated a significant increase in Cav-1 mRNA and protein expression in the RCI group compared to the Control group (Fig. 1B, C), suggesting a potential essential role for Cav-1 in the pathogenesis of RCI.

Furthermore, we employed Western blot technology to assess the expression of fatty acid synthesis-related protein (FASN) and peroxisome proliferator-activated receptor-gamma (Pparg). The findings revealed a significant upregulation of FASN and Pparg expression in the RCI group relative to the control group (Fig. 1D). In summary, these findings enhance our understanding of the role of Cav-1 in RCI and also shed light on the potential importance of the adipogenesis process in RCI pathology.

### Unveiling cell phenotypic changes in RCI using scRNA-seq

**Fig. 2:**
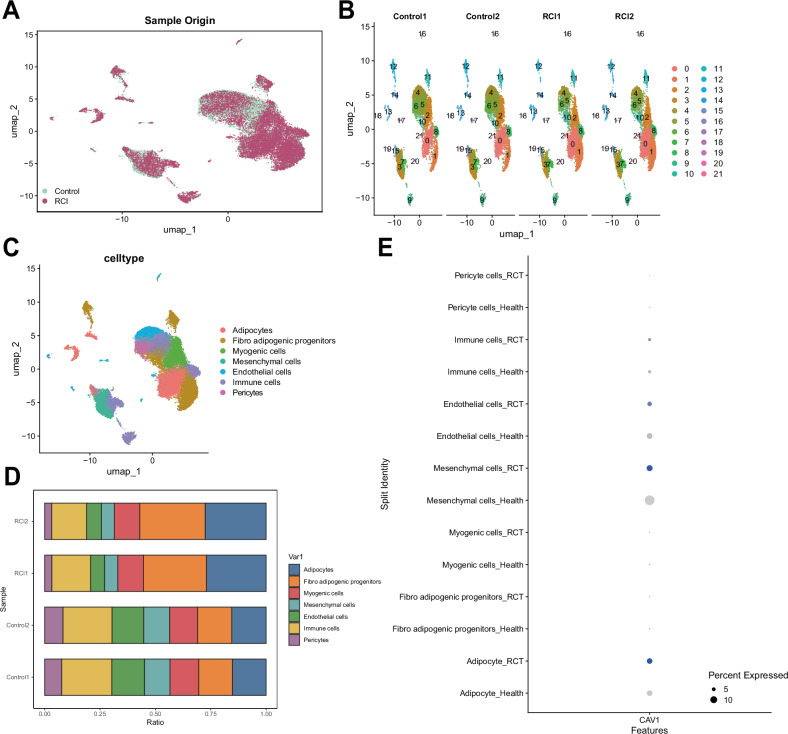
Cell clustering and annotation of scRNA-seq data. **A** UMAP visualization of clustered and distributed cells in tissue samples from Control (*n* = 2) and RCI (*n* = 2) sources in a two-dimensional representation. **B** UMAP visualization of clustered and distributed cells in samples from different sources, with each color representing a cluster. **C** UMAP-based visualization of cell annotation results, with each color representing a cell population. **D** Stacked bar chart showing the expression proportions of different cell types in the Control and RCI groups, where each color represents a cell type and the width represents its proportion in the samples. **E** Differential expression of the Cav-1 gene in the scRNA-seq data between the Control group (*n* = 2) and the RCI group (*n* = 2). The size of the dots indicates the percentage of cells expressing the gene within each cell group (25%, 50%, 75%), and the shade of blue represents the expression level of the gene, with deeper blue indicating higher expression.

We processed and integrated the data using the Seurat package, identifying 22 cell types and observing significant changes in cell phenotypes among samples from different sources (Fig. 2A, B). UMAP dimensionality reduction analysis revealed the distribution and clustering of different cell populations in high-dimensional expression data, identifying seven cell types (Fig. 2C): Adipocytes, Fibro-adipogenic progenitors, Myogenic cells, Mesenchymal cells, Endothelial cells, Immune cells, and Pericytes. We compared their variations between the Control and RCI groups, finding that the proportion of Adipocytes was significantly higher in the RCI group (Fig. 2D). We also evaluated the cell marker factors, where PPARG indicated Adipocytes, PDGFRA and CD34 indicated Fibro-adipogenic progenitors, MYF6 indicated Myogenic cells, PECAM1 indicated Endothelial cells, CD4 indicated Immune cells, PDGFRB indicated Pericytes, and S100A4 indicated Mesenchymal cells (Fig. S1A). Additionally, cell communication analysis showed significantly enhanced interactions between Adipocytes and Fibro-adipogenic progenitors, Mesenchymal cells, and Immune cells in the RCI group (Fig. S1B, C), suggesting the important role of Adipocytes in the occurrence of RCI.

Furthermore, we examined the expression of Cav-1 across various cell subpopulations, and the results showed that Cav-1 expression was significantly higher in the RCI group compared to the Control group, particularly in Adipocytes (Fig. 2E).

These results indicate that the proportion of Adipocytes significantly increases in the RCI group, with higher expression of Cav-1 in these cells compared to the Control group, and enhanced communication with various cell types. This suggests the critical role of Adipocytes and Cav-1 in the process of RCI development.

### Dynamic infiltration of adipocytes in muscle tissue of RCI patients revealed by temporal analysis

**Fig. 3:**
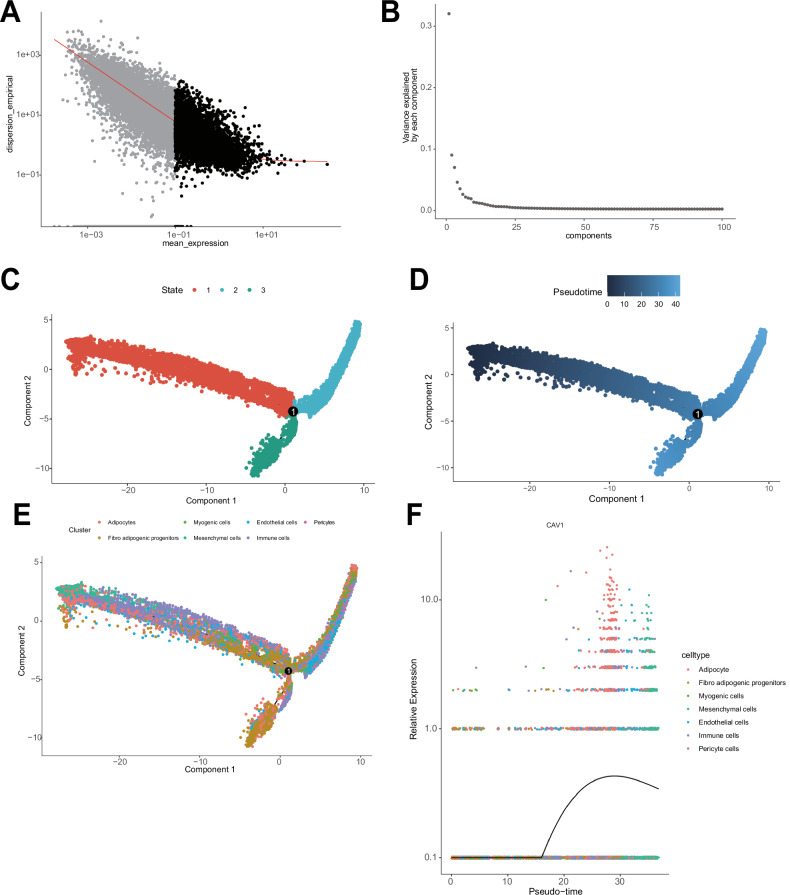
Temporal analysis of scRNA-seq data. **A** Visualization of sorting genes, each dot represents a gene. **B** PCA, where the *x*-axis represents the component number and the *y*-axis represents the proportion of variance explained. **C** Trajectory skeleton plot displaying cellular differentiation states in different branches. **D** Trajectory skeleton plot showing pseudo-temporal analysis trajectory of adipocyte differentiation. **E** Different colors represent the distribution states of different cell types along the pseudo-temporal trajectory. **F** Pseudotime analysis showing the trend of Cav-1 gene expression over pseudotime.

Temporal analysis further revealed the dynamic infiltration of adipocytes in the muscle tissue of RCI patients. We assessed the infiltration of adipocytes in the muscle tissue using DDRTree for data dimensionality reduction. The visualization of sorted genes using PCA showed the complexity and dimensionality of the dataset (Fig. 3A). Additionally, analysis of the gene expression trends revealed their contribution to data variability (Fig. 3B). The distribution and variation of different cells along the pseudo-time trajectory, representing their state and pseudo-time, were also demonstrated (Fig. 3C, D), highlighting the dynamic changes in cell state over time. Based on the gradient of pseudo-time, we inferred a continuous process of cell differentiation and maturation. Notably, adipocytes exhibited high expression across all three trajectories of cell state evolution. Furthermore, fibro-adipogenic progenitors, which have the potential to differentiate into adipocytes, showed significantly increased expression at the terminal differentiation phase in the pseudo-time analysis (Fig. 3E) [19]. The heightened activity of these multi-potent progenitor cells, along with an increase in adipocyte numbers, may contribute to fat infiltration or fibrosis in the muscle tissue post-RCI, potentially impeding muscle function recovery. Additionally, the curve of Cav-1 gene expression over Pseudotime indicates that as Pseudotime progresses, the expression of Cav-1 significantly increases, particularly in Adipocytes (Fig. 3F), suggesting the dynamic role of Cav-1 in the development of RCI. In summary, these findings uncover a potential association between adipocyte heterogeneity in muscle tissue and the RCI process.

### Cav-1 plays a critical role in 3T3-L1 adipocyte differentiation

**Fig. 4:**
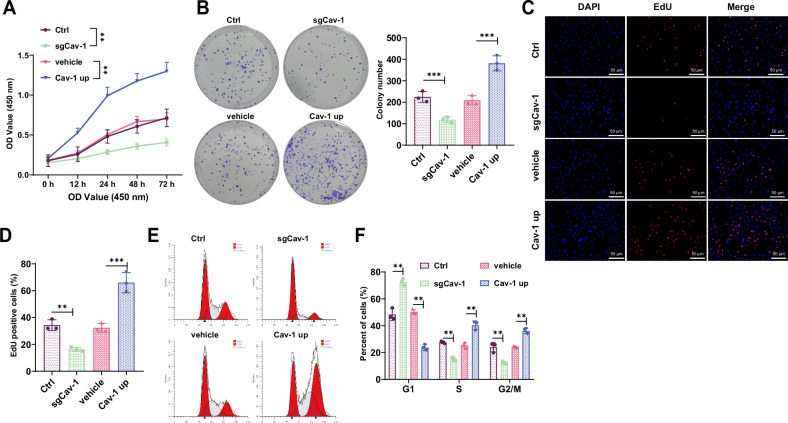
Regulation of 3T3-L1 cell differentiation by Cav-1. **A** CCK8 assay assessing the viability of 3T3-L1 cells in each group. **B** Colony formation assay examining the clonogenic ability of 3T3-L1 cells in each group. **C**, **D** Immunofluorescence staining to assess the proliferation of 3T3-L1 cells in each group, Scale bar = 50 μm. **E**, **F** Flow cytometry to examine the cell cycle distribution of 3T3-L1 cells in each group. *** indicates *P* < 0.001, ** indicates *P* < 0.01. Cell experiments were performed in triplicate.

In this study, we found a close association between Cav-1 and RCI. To further explore the correlation between Cav-1 and the adipogenic differentiation process of 3T3-L1 cells, experiments were conducted on 3T3-L1 cells cultured in vitro. At different time points (D0, D7, D17), Oil Red O staining was performed to assess the adipogenic differentiation capacity of the cells. The results indicated that the ability of 3T3-L1 cells to differentiate into adipocytes increased over time (Fig. S2A). Simultaneously, RT-qPCR and Western blot analyses were employed to examine the expression of Cav-1. The findings showed a significant elevation in Cav-1 mRNA and protein levels, demonstrating a time-dependent increase correlated with the adipogenic differentiation process of 3T3-L1 cells (Fig. S2B, C). Furthermore, the CRISPR-Cas9 technology was utilized to knock out the Cav-1 gene (Fig. S2D), and a Cav-1 overexpression cell model was constructed using lentiviral transfection in 3T3-L1 cells (Fig. S2E). CCK8 assay results demonstrated a significant decrease in cell viability of SgCav-1 group 3T3-L1 cells compared to the Ctrl group, while the overexpression of Cav-1 in 3T3-L1 cells (Cav-1 up) showed enhanced cell viability compared to the vehicle group (Fig. 4A). Colony formation assay further validated these findings, as the number of colonies formed by SgCav-1 group 3T3-L1 cells significantly decreased compared to the Ctrl group, indicating a reduction in clone formation. Conversely, the Cav-1 up group 3T3-L1 cells exhibited a significant increase in colony numbers, suggesting an improvement in clone formation ability (Fig. 4B).

Furthermore, the proliferation of 3T3-L1 cells was evaluated using EdU immunofluorescence staining. The results showed a significant decrease in proliferation capacity of SgCav-1 group 3T3-L1 cells compared to the Ctrl group, while the Cav-1 up group 3T3-L1 cells displayed an upregulation in proliferation ability (Fig. 4C, D). Cell cycle distribution was analyzed using flow cytometry. Compared to the Ctrl group, SgCav-1 group 3T3-L1 cells exhibited an increased proportion in the G1 phase and a decreased proportion in the S and G2/M phases, indicating cell cycle inhibition. In contrast, the Cav-1 up group 3T3-L1 cells showed a decrease in the G1 phase proportion and a significant increase in the G2/M phase proportion, indicating that Cav-1 overexpression promotes cell cycle progression (Fig. 4E, F).

In summary, these findings suggest a significant role of Cav-1 in the proliferation and differentiation of 3T3-L1 cells. The expression of Cav-1 significantly affects the viability, proliferation, and cell cycle progression of 3T3-L1 cells, highlighting its crucial involvement in regulating the biological behavior of adipocytes.

### Cav-1 regulates adipocyte differentiation and lipid metabolism

**Fig. 5:**
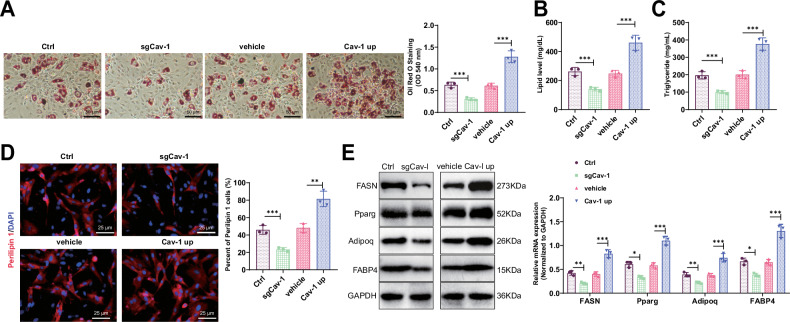
Cav-1 regulates lipid accumulation and adipogenesis in myocytes. **A** Oil Red O staining to assess the differentiation capacity of 3T3-L1 cells in each group, Scale bar = 50 μm. **B** Expression analysis of lipid in each group of 3T3-L1 cells. **C** Expression analysis of Triglycerides in each group of 3T3-L1 cells. **D** Immunofluorescence staining to examine the expression of Perilipin 1 in each group of 3T3-L1 cells, Scale bar = 25 μm. **E** Western blot analysis of FASN, Pparg, Adipoq, and FABP4 expression in each group of 3T3-L1 cells. *** indicates *P* < 0.001, ** indicates *P* < 0.01, * indicates *P* < 0.05. Cell experiments were performed in triplicate.

In this study, we investigated the impact of Cav-1 on lipid accumulation and adipogenesis in 3T3-L1 cells. Quantitative results from Oil Red O staining revealed the influence of Cav-1 on the differentiation of 3T3-L1 cells into mature adipocytes. The findings demonstrated that the lipid differentiation capacity of 3T3-L1 cells was decreased in the SgCav-1 group compared to the Ctrl group, while it was enhanced in the Cav-1 up group compared to the vehicle group (Fig. 5A). Additionally, we assessed the levels of Lipids and Triglycerides. Compared to the Ctrl group, the expression of Lipids and Triglycerides in 3T3-L1 cells of the SgCav-1 group was downregulated, whereas the overexpression of Cav-1 in the 3T3-L1 cells of the Cav-1 up group significantly increased the expression of Lipids and Triglycerides (Fig. 5B, C). This suggests that Cav-1 may promote lipid synthesis and storage.

The expression of the mature adipocyte marker Perilipin 1 was detected through immunofluorescence staining. Results showed a significant decrease in the number of Perilipin 1-positive cells in the SgCav-1 group of 3T3-L1 cells compared to the Ctrl group, while the expression of Perilipin 1 in the 3T3-L1 cells of the Cav-1 up group demonstrated the opposite trend (Fig. 5D). Western blot analysis further confirmed the critical role of Cav-1 in adipose biosynthesis. The results revealed that FASN, Pparg, Adipoq, and FABP4 expression, which are proteins involved in fat generation, were downregulated in the SgCav-1 group compared to the Ctrl group. Conversely, the Cav-1 up group of 3T3-L1 cells exhibited an increase in the expression of the aforementioned proteins (Fig. 5E), indicating that Cav-1 activates adipocyte differentiation and fat synthesis.

In summary, our study demonstrates that Cav-1 is a crucial factor in regulating adipocyte differentiation and lipid metabolism. By modulating the expression of Cav-1, we can significantly influence lipid accumulation and adipogenesis in 3T3-L1 cells.

### Differential gene expression analysis reveals Cav-1’s role in adipogenesis and lipid metabolism

**Fig. 6:**
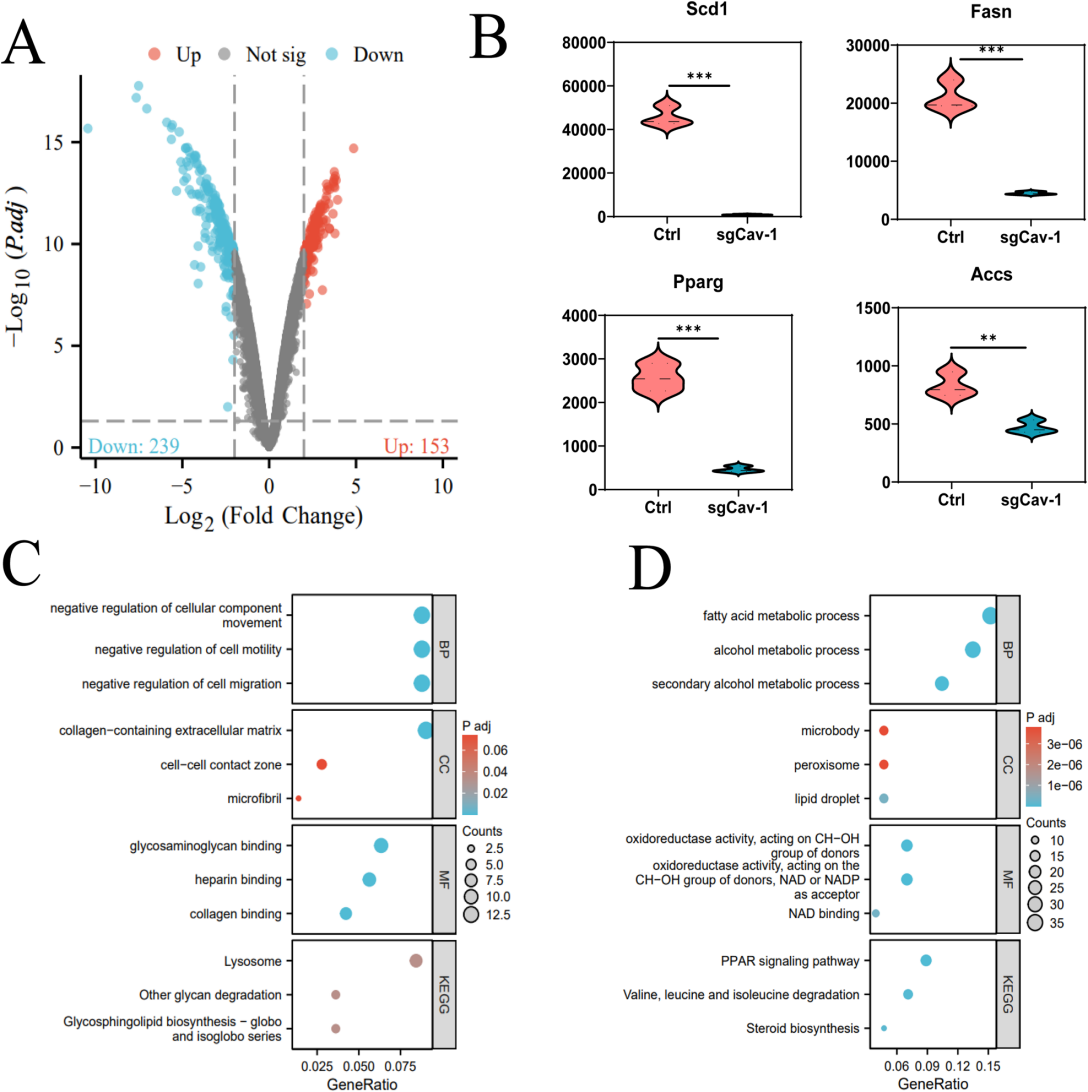
Transcriptome sequencing analysis reveals the impact of Cav-1 deficiency on the expression of adipogenesis-related genes. **A** Differential gene expression analysis of Ctrl group and SgCav-1 group 3T3-L1 cells using transcriptome sequencing. Blue dots represent downregulated genes, red dots represent upregulated genes, and gray dots represent non-significant genes. The experiment was repeated three times. **B** Expression levels of Scd1, FASN, Pparg, and Accs in the transcriptome sequencing results. **C** KEGG and GO analyses of upregulated genes in the transcriptome sequencing analysis. **D** KEGG and GO analyses of downregulated genes in the transcriptome sequencing analysis. *** indicates *P* < 0.001, ** indicates *P* < 0.01.

Based on the aforementioned research, we have discovered a close association between Cav-1 and lipid accumulation and adipogenesis in 3T3-L1 cells. In order to further investigate the mechanism by which Cav-1 regulates adipocyte differentiation in 3T3-L1 cells, we performed high-throughput transcriptome sequencing to analyze the DEGs between the Ctrl group and the SgCav-1 group of 3T3-L1 cells. Our results identified a total of 239 downregulated genes and 153 upregulated genes (Fig. 6A). Notably, compared to the Ctrl group, the SgCav-1 group exhibited significantly decreased expression of genes, such as Scd1, FASN, Pparg, and Accs, which are closely related to fatty acid synthesis (Fig. 6B). These findings indicate that the loss of Cav-1 disrupts the function and development of adipocytes.

Furthermore, we conducted a KEGG pathway and GO functional enrichment analysis to gain insight into the potential functions of these DEGs. Among the upregulated genes, the biological processes primarily involved negative regulation of cellular component movement, cell motility, and cell migration, as well as the biological pathways of Lysosome and Other glycan degradation (Fig. 6C). On the other hand, the downregulated genes were found to be significantly associated with signal pathways related to fatty acid metabolism, regulation of lipid metabolic process, and triglyceride metabolic process, including Pparg, Dbi, Acss2, FASN, Scd1, and Adipor2 (Figs. 6D, S3).

In conclusion, our transcriptome sequencing analysis provides further evidence of the critical role of Cav-1 in regulating adipocyte differentiation and lipid metabolism. The downregulation of Cav-1 leads to a significant decrease in the expression of crucial genes involved in adipocyte differentiation, thereby inhibiting lipid accumulation and impairing adipocyte function.

### Exercise reduces fat infiltration in RC repair by regulating Cav-1

First, using immunofluorescence staining techniques, we evaluated the Cav-1 levels in different experimental groups at the 4th and 8th weeks after surgery in RC sites. The results showed that compared to the Sham group, the Model group had significantly increased Cav-1 levels at the 4th and 8th weeks in the RC sites. However, after treatment with the Treadmill group after surgery, we observed a significant decrease in Cav-1 expression compared to the Model group (Fig. S4A, B). Western blot experiments further confirmed the above results, with the Model group showing significantly increased levels of Cav-1 expression compared to the Sham group. Compared to the Model group, the Treadmill group exhibited decreased Cav-1 expression levels, which further decreased over time (Fig. S4C, D), highlighting the significant inhibitory effect of exercise on Cav-1 expression levels in RC sites.

**Fig. 7:**
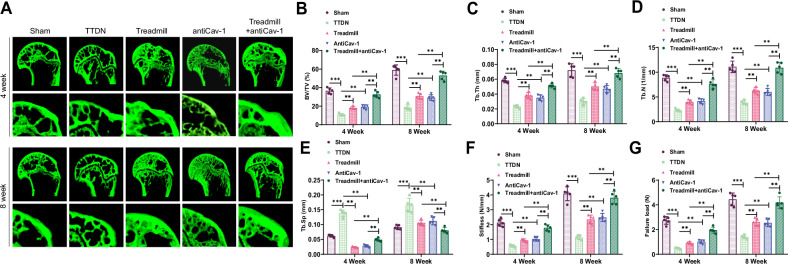
SR-μCT analysis at different time periods after RCI surgery. **A** Representative SR-μCT reconstruction images of the healing sites at the 4th and 8th weeks after surgery in each group. **B**–**E** Results of BV/TV (**B**), TB/Th (**C**), TB/N (**D**), and TB/sp (**E**) at different time points after RC injury repair in each group. **F**, **G** Biomechanical testing results showing the stiffness and failure load of the supraspinatus tendon-humerus complex in mice. *** indicates *P* < 0.001, ** indicates *P* < 0.01, and *n* = 5 in animal experiments.

Next, we used high-resolution synchrotron radiation micro-computed tomography (SR-μCT) technology to perform 3D reconstructions of the RC healing sites at weeks 4 and 8 post-surgery to quantitatively assess the microstructural parameters of new bone (Fig. 7A). These parameters included BV/TV, Tb.Th, Tb.N, and Tb.Sp, collectively reflecting the quality and structural integrity of the new bone during the healing process. The results showed that compared to the Sham group, the Model group had decreased BV/TV, Tb.Th, and Tb.N, while Tb.Sp significantly increased, indicating that tendon detachment had a significant impact on the structure and quality of the new bone. Interestingly, compared to the Model group, the aforementioned parameters improved in the Treadmill and antiCav-1 groups, suggesting that treadmill treatment may promote healing at the RC site. Notably, compared to the Treadmill or antiCav-1 groups, the Treadmill+antiCav-1 group showed even more significant improvements in the microstructural parameters of new bone (Fig. 7B–E), indicating the crucial role of Cav-1 in treadmill-induced RC repair and suggesting that inhibition of Cav-1 may further enhance new bone formation and healing.

To further evaluate tendon healing, we conducted biomechanical tests on supraspinatus tendon-humerus complexes from the five groups of mice, comparing the stiffness and failure load. The results showed that the stiffness and failure load of the supraspinatus tendon-humerus complex in the Model group were significantly lower than those in the Sham group. Compared to the Model group, the stiffness and failure load increased in the Treadmill and antiCav-1 groups, indicating that treadmill or antiCav-1 treatment might promote healing at the RC site. Moreover, compared to the Treadmill group, the Treadmill+antiCav-1 group showed a significant increase in stiffness and failure load (Fig. 7F, G).

Overall, our study emphasizes the dual role of Cav-1 in RC injury repair and new bone formation, and it highlights the significant improvement effect of exercise in promoting the healing process through the inhibition of Cav-1 expression.

### The functional role of Cav-1 in muscle fat infiltration and repair

This study aims to investigate the functional role of Cav-1 in fat infiltration in RCI muscle, as well as its correlation with fat deposition during muscle repair. Our experimental results reveal a significant association between Cav-1 levels and muscle loss and tissue repair quality, providing new biomarkers for understanding RCI repair.

**Fig. 8:**
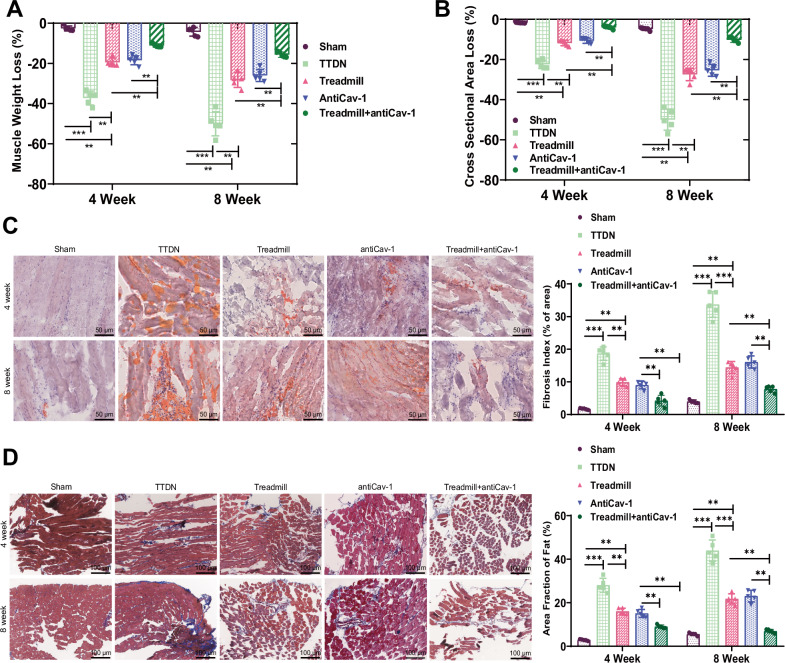
Cav-1 regulates lipid accumulation and adipogenesis in muscle fat cells. **A** Percentage decrease in muscle weight in different groups of mice. **B** Percentage of cross-sectional area of muscle fibers in different groups of mice. **C** Oil Red O staining showing fat infiltration in muscle of different groups of mice. **D** Masson’s trichrome staining revealing collagen protein expression in muscle of different groups of mice.*** indicates *P* < 0.001, ** indicates *P* < 0.01, and *n* = 5 in animal experiments.

First, we measured the muscle weight and cross-sectional area of the SS muscle in different groups of mice to quantify muscle atrophy. The results showed that, compared to the Sham group, the Model group exhibited a significant decrease in muscle weight and an increase in cross-sectional area at 4 and 8 weeks post-surgery. In comparison, the Treadmill and antiCav-1 group showed a slight increase in muscle weight and a deceleration in the increase of cross-sectional area. Furthermore, the Treadmill + antiCav-1 group exhibited a more significant increase in muscle weight and a more noticeable decrease in cross-sectional area (Fig. 8A, B), indicating the important role of inhibiting Cav-1 in muscle injury and repair processes.

Using Oil Red O and Masson’s trichrome staining, we further investigated fat infiltration and collagen protein expression in the SS muscle of mice in each group. The results showed that compared to the Sham group, the Model group exhibited increased fat infiltration and collagen deposition. In comparison, the Treadmill and antiCav-1 group showed a decrease in fat infiltration and collagen protein. Additionally, the Treadmill + antiCav-1 group exhibited a more pronounced recovery of muscle weight and a reduction in cross-sectional area, especially over time, leading to a more evident decrease in fat deposition and fibrosis (Fig. 8C, D).

Moreover, Western blot analysis was performed to examine the expression of FASN and Pparg (related to fat synthesis) and ATGL and HSL (related to protein degradation). The results demonstrated that in comparison to the Sham group, the Model group exhibited a significant increase in FASN and Pparg expression and a significant downregulation in ATGL and HSL expression. In contrast, the Treadmill and antiCav-1 group displayed a significant downregulation in FASN and Pparg expression and an upregulation in ATGL and HSL expression. Furthermore, the Treadmill + antiCav-1 group showed superior regulation of the mentioned proteins compared to the Treadmill and antiCav-1 group (Fig. S5A, B), further supporting the critical role of Cav-1 in regulating fat synthesis and metabolism.

In conclusion, our study reveals the involvement of Cav-1 in muscle fat infiltration and fibrosis and suggests that it may affect muscle quality and functional recovery by regulating the expression of fat synthesis and degradation enzymes.

## Discussion

RCI is a common shoulder condition, particularly among middle-aged and elderly individuals, involving damage or inflammation of the RC tendons. The etiology of RCI is multifactorial, potentially stemming from chronic overuse, traumatic injury, or age-related degenerative changes [20]. Clinically, RCI presents with shoulder pain and joint dysfunction, which, in severe cases, can significantly impact a patient’s quality of life [21]. Fat infiltration plays a crucial role in the progression of RCI [22, 23]. Previous studies have identified certain molecular mechanisms, such as the mediation by inflammatory factors and cytokines, that regulate fat infiltration in RCI [24, 25]. However, few studies have combined the regulation of exercise and Cav-1 to explore their role in mitigating fat infiltration in RCI [26]. This study stands out by using molecular biological analysis to reveal the significant role of exercise and Cav-1 in inhibiting fat cell infiltration.

Previous studies have revealed communication patterns between fat cells and other cell populations [27–29]. However, these studies often lacked a comprehensive exploration of cellular heterogeneity in RCI tissue [3, 5]. In this study, scRNA-seq was utilized to unveil cell subpopulations’ heterogeneity in RCI tissue, with a particular focus on the intercellular communication between fat cells and other cell populations. This finding aids in a deeper understanding of different cell types’ functions in RCI and their regulatory roles in fat infiltration.

Cav-1 has been shown to play a critical role in adipocyte differentiation in previous studies [30, 31]. The results of this study further corroborate this point. By knocking out the Cav-1 gene, we observed a significant inhibition of adipocyte differentiation-related gene expression, which plays a key role in lipid metabolism. This finding reveals the crucial role of Cav-1 in the fat infiltration process of RCI.

Compared to previous research, this study unravels the regulatory mechanism of exercise on Cav-1 expression. By combining single-cell sequencing techniques with in vitro and in vivo experimental evidence, we revealed that exercise exerts a significant impact on improving fat infiltration in RCI through the regulation of Cav-1 expression. These findings further confirm the positive influence of exercise in RCI and highlight Cav-1 as a potential regulatory factor.

RCI can indeed be caused by various factors, including trauma, chronic overuse, and age-related degenerative changes, which may introduce certain limitations when using samples from trauma patients. The pathological features of traumatic RCI may differ from those of other types of RCI, particularly in terms of molecular characteristics related to cell repair, inflammatory response, and fat infiltration. Therefore, the findings of this study are highly applicable to traumatic RCI patients, but they may show different molecular regulatory patterns in cases of chronic overuse or degenerative RCI. This limitation should be noted in the discussion, and caution should be exercised when generalizing the results to a broader RCI patient population to avoid inappropriate extrapolation. Future studies should consider including other types of RCI patients to more comprehensively evaluate the role of Cav-1 and its molecular regulatory mechanisms in RCI caused by different etiologies. This approach would help further validate the conclusions of this study and enhance its applicability to a more diverse patient population.

Compared to other non-surgical treatments such as medication and physical therapy, exercise intervention has unique effects on fat infiltration in RCI. By modulating the expression of Cav-1, exercise intervention can improve fat infiltration in RCIs. This finding provides new molecular biology evidence for non-surgical treatments of RCI and offers potential targets for developing relevant therapies.

The scientific value of this study is revealed in its demonstration of the regulatory role of exercise in improving fat infiltration in RCI. Through analysis of intercellular communication patterns and gene function enrichment, the study clarifies the critical role of Cav-1 in adipocyte differentiation and lipid metabolism. This provides new molecular biology evidence for understanding the pathogenesis of RCI and the feasibility of non-surgical treatments. By uncovering how exercise modulates the expression of Cav-1 to further reduce fat infiltration in the RCI area, this study provides new therapeutic targets for RCI rehabilitation. Additionally, the study uses scRNA-seq to analyze cellular heterogeneity and the communication patterns between adipocytes and other cell populations in RCI tissues, which provides a more detailed and comprehensive understanding of RCI’s occurrence and development. Moreover, in vivo and in vitro experiments validate the regulatory role of Cav-1 in adipocyte differentiation and further confirm that exercise can reduce fat infiltration and promote structural repair in the injury area. These experimental results lay the foundation for further exploration of clinical applications of related treatment strategies.

Based on existing findings, we hypothesize that during the early repair phase of RCI, Cav-1 primarily influences fat infiltration in the injured area by regulating the differentiation and proliferation of adipocytes. High early expression of Cav-1 can inhibit the maturation of adipocyte precursors and reduce the accumulation of adipocytes, thereby alleviating fat infiltration at the injury site. This process helps protect the muscle structure of the RC region, maintain tissue elasticity and strength, and facilitate the initiation of cell migration and repair. In the later repair phase of RCI, the regulation of Cav-1 expression becomes more complex. As tissue remodeling occurs, Cav-1 participates in the interactions between fibroblasts and muscle cells, which is crucial for reducing fibrosis and restoring tissue elasticity. However, excessively high or abnormal Cav-1 expression may exacerbate fat infiltration and fibrosis, hindering long-term repair and functional recovery. Therefore, moderate Cav-1 expression can inhibit further differentiation of adipocytes in the late phase, helping to reduce irreversible fat infiltration and fibrosis. Overall, Cav-1 can alleviate fat infiltration in the early phase of RCI by reducing adipocyte differentiation, while in the later phase, appropriate regulation is necessary to balance fibrosis and adipogenesis, providing a favorable microenvironment that supports the dynamic equilibrium during RCI repair. This suggests targeted Cav-1 molecular regulation strategies for different RCI stages.

However, this study also has some limitations. First, the analysis is based only on shoulder cuff tissue samples from RCI patients. It would be beneficial to increase the sample size and conduct more in-depth research on patients at different clinical stages. Second, although exercise can reduce fat infiltration and promote structural repair in the injury area by modulating Cav-1 expression, further optimization of treatment plans and evaluation of long-term effects and safety are necessary for practical clinical application. When designing and analyzing animal experiments, gender and age are critical biological variables, as differences in metabolism, hormone levels, and cell signaling pathways can significantly impact experimental results. In particular, when studying adipocyte differentiation and Cav-1 expression, hormonal differences between genders (e.g., estrogen and testosterone) may modulate the responsiveness of adipose tissue, thus influencing the degree of fat infiltration and the sensitivity to exercise intervention. Additionally, with aging, the characteristics and differentiation potential of adipose tissue change, with older animals potentially showing reduced responsiveness to exercise, while younger animals have greater adaptive capacity. Therefore, future experiments should consider or control these variables to enhance the generalizability of results and avoid biases when applying findings to different age and gender groups.

Based on bioinformatics analysis and in vitro and in vivo experimental results, this study demonstrates that modulating Cav-1 expression significantly affects lipid accumulation and adipogenesis in 3T3-L1 cells. We revealed the role of Cav-1 in muscle fat infiltration and fibrosis, showing that exercise regulates Cav-1 expression, thereby modulating the molecular mechanisms of adipocyte differentiation and improving fat infiltration in RC injuries at the molecular level. As a crucial membrane protein, Cav-1 plays a key role in cell signal transduction and lipid metabolism, and its potential regulatory effects on other key cell groups involved in injury repair warrant further investigation. First, the differential expression of Cav-1 in tenocytes and its silencing or overexpression may impact tenocyte proliferation and differentiation, contributing to tendon regeneration and repair. Second, Cav-1’s regulation in immune cells is also critical. It has been shown to modulate macrophage IL-1β secretion and cell death to maintain appropriate immune responses [32]. Immune cells are not only involved in the inflammatory response but also play significant roles in tissue remodeling during injury repair [33, 34]. Additionally, Cav-1 can regulate the polarization state of macrophages, influencing the intensity of the inflammatory response, with studies indicating that excessive Cav-1 expression may lead to macrophage hyperactivation and dysregulated inflammation [35].

**Fig. 9:**
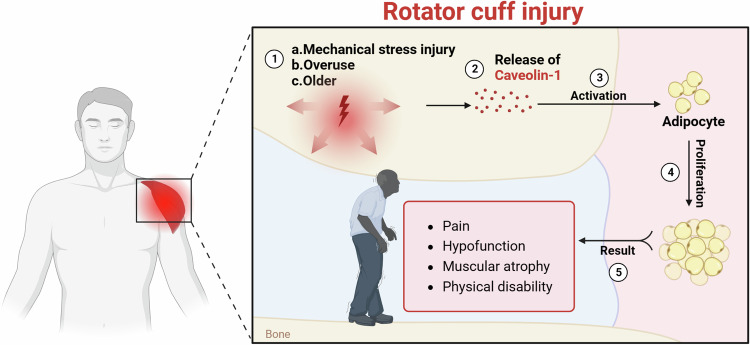
Exercise improves RCI fat infiltration by regulating Cav-1 expression revealed by single-cell sequencing.

In conclusion, this study highlights the regulatory role of Cav-1 in tenocytes and immune cells, as well as its potential impact on the local microenvironment and intercellular interactions, contributing to the modulation of the overall injury repair process. Additionally, our findings demonstrate that exercise can improve fat infiltration in RC injuries by regulating Cav-1 expression, providing new molecular insights and potential targets for non-surgical treatment. Future research should further explore the specific mechanisms of Cav-1 and its application in RCI rehabilitation, as well as optimize exercise intervention strategies to enhance patient recovery. Despite certain limitations, this study offers new directions and perspectives for the treatment and rehabilitation of RC injuries, with the hope of making significant contributions to future clinical applications (Fig. 9).

## Materials and methods

### Clinical sample collection

Samples from 18 patients with RCI were collected at our hospital between January 2023 and December 2023. Post-surgery, supraspinatus tendon tissues were obtained from the injured rotator cuffs of these patients (RCI group) and were stored at temperatures below −80 °C for subsequent molecular biological analysis. Additionally, shoulder cuff tissue from 18 age- and consultation time-matched patients with proximal humeral fractures were selected as the control group (Control group). Radiological evaluations (X-ray and ultrasound) confirmed that these control patients had no pre-existing shoulder pain, history of RC tears, or any clinical signs indicative of such conditions [36] (Table S1). Prior to clinical resource collection, all patients had provided informed consent, and the study had been approved by the institutional ethics committee, adhering to the principles outlined in the Declaration of Helsinki.

The inclusion criteria for the RCI group were as follows: (1) age ≥18 years; (2) trauma-induced shoulder dysfunction confirmed by MRI and accompanied by clinical symptoms of RCI; (3) fully repairable tears (coverage >80%, with no tension issues confirmed during surgery); and (4) RCI classified as partial-thickness or full-thickness tears [37].

### Single-cell sequencing and data analysis

Tissue samples from the postoperative shoulder cuff of both the aforementioned Control group (*n* = 2) and RCI group (*n* = 2) were collected. They were processed into single-cell suspensions using trypsin (9002-07-7, Sigma-Aldrich, USA). Single cells were captured using the C1 Single-Cell Auto Prep System (Fluidigm, Inc., South San Francisco, CA, USA), where the cells were lysed, releasing mRNA, which was then reverse transcribed into cDNA. The lysed and reverse-transcribed cDNA underwent pre-amplification in a microfluidic chip for subsequent sequencing. The amplified cDNA was used to construct libraries, and single-cell sequencing was performed on the HiSeq 4000 Illumina platform (parameters: paired-end reads, read length of 2 × 75 bp, approximately 20,000 reads per cell) [38].

The data were analyzed using the “Seurat” package in R software. First, a series of quality control measures were applied to the data with filter thresholds set as nFeature_RNA > 500, nCount_RNA > 1000, nCount_RNA < 20,000, and percent.mt < 5. Batch effects in the data were removed by Canonical Correlation Analysis (CCA), followed by standardization with the LogNormalize function. Principal component analysis (PCA) was conducted, and relevant components were selected for t-distributed stochastic neighbor embedding (t-SNE) clustering analysis. Markers for various cell subtypes were identified using the Seurat package, and annotation of marker genes for each cell cluster was performed using the “SingleR” package [39]. Cell communication analysis was conducted using the “CellChat” package in R. Finally, pseudotime analysis was performed using the “monocle” package, and data dimensionality reduction was achieved using DDRTree. Cells were sorted, and trajectories were constructed based on the expression trends of ordered genes [39, 40].

### Transcriptome sequencing and data analysis

Three replicates of Ctrl and sgCav-1 groups of 3T3-L1 cells were collected for high-throughput transcriptome sequencing to ensure the accuracy of the data. The specific steps were as follows: total RNA from each sample was extracted using Trizol reagent (T9424, Sigma-Aldrich, USA) according to the manufacturer’s instructions. The concentration, purity, and integrity of the RNA were measured using the Qubit®2.0 Fluorometer® (Life Technologies, CA, USA) with the Qubit®RNA Assay Kit, the NanoDrop spectrophotometer (IMPLEN, California, USA), and the RNA Nano 6000 Assay Kit on the Bioanalyzer 2100 system (Agilent Technologies, Santa Clara, California, USA). The A260/280 ratio should be between 1.8 and 2.0. The total RNA content of each sample was 3 μg, which served as the input material for RNA sample preparation. Following the manufacturer’s recommendations, the NEBNext® UltraTM RNA Directional Library Prep Kit (E7760S, New England Biolabs, China) suitable for Illumina was used to generate cDNA libraries, and the quality was assessed on the Agilent Bioanalyzer 2100 system. The samples were indexed and clustered using the TruSeq PE Cluster Kit v3 cBot HS (Illumina) on the cBot cluster generation system. After cluster generation, library sequencing was performed on the Illumina-Hiseq 550 platform, generating paired-end reads of 125 bp/150 bp [41].

After obtaining the gene expression matrix, differential analysis was performed using the limma package in R software, with a threshold of |log2FC| > 2 and *P* < 0.05, to filter differentially expressed genes (DEGs). The “pheatmap” package in R software was used to generate volcano plots of DEG expression [42].

### Gene functional enrichment analysis

We conducted Kyoto Encyclopedia of Genes and Genomes (KEGG) and Gene Ontology (GO) pathway enrichment analysis (setting *P* < 0.05) using the R software package clusterProfiler to analyze the overlapped genes obtained. Furthermore, we generated bubble plots for biological processes (BP), cellular components (CC), and molecular functions (MF) within the GO category [43].

### Cell culture and treatment

The 3T3-L1 preadipocytes (CL-173, ATCC) were seeded in a 6-well plate and grown in Dulbecco’s Modified Eagle Medium (12491015, Gibco) supplemented with 10% fetal bovine serum (FBS) (12106 C, Sigma) and 1% penicillin/streptomycin (15140122, Gibco). The cells were incubated in a CO_2_ incubator at 37 °C for 48 h. Subsequently, the cells were induced to differentiate by incubating them in DMEM supplemented with 10% FBS, 1 μg/mL insulin (P3376-100IU, Beyotime), 1 μM dexamethasone (ST1254, Beyotime), and 0.5 mM IBMX (I5879, Sigma) for 48 h. On day 7 (D7) and day 14 (D14), cells from each group were harvested for further analysis [44].

### CRISPR/Cas9 gene editing technique

Cav-1 knockout (sgCav-1) cells were generated using the CRISPR/Cas9 technology. The sgRNA design used was provided by Synthego (USA) and had the following sequence: Cav-1-sgRNA: Forward: 5’-ATCTCTACACTGTTCCCATC-3’, Reverse: 5’-AUCUCUACACUGUUCCCAUC-3’. The sgRNA was inserted into the Lenti-CRISPR v2 vector (HanBio, Shanghai, China) containing the Streptococcus pyogenes Cas9 nuclease gene. Cells were transduced with the lentiviral Lenti-CRISPR v2 vector to generate sgCav-1 cells using the CRISPR/Cas9 editing system. Cells transfected with sgRNA plasmids and donor sequences were selected using 4 μg/mL puromycin (HY-K1057, MCE, USA). Screening methods, including restriction dilution cloning and Western blot analysis, were used to identify sgCav-1 cells [45, 46].

### Lentivirus

Cav-1 overexpression cell lines (Cav-1 up) and corresponding control cell lines (vehicle) were constructed using lentivirus transduction. The plasmids carrying the Cav-1 overexpression gene, along with the helper plasmids Pspax2 and Pmd2.G, were transfected into 293T cells (CRL-3216, ATCC). Lentivirus was collected within 48 h after transfection. Plasmids and lentivirus packaging services were provided by Shenggong Life Sciences (Shanghai, China).

For lentivirus-mediated transfection of 3T3-L1 preadipocytes, 1 × 10^5^ cells were seeded in 6-well plates. When the cell confluence reached 70–90%, the culture medium containing an appropriate amount of packaged lentivirus (MOI = 10, working titer approximately 1 × 10^6^ TU/mL) and 5 μg/mL polybrene (TR-1003, Sigma-Aldrich) was added for transfection. After 4 h of transfection, an equal amount of fresh medium was added to dilute the polybrene, and then the medium was replaced 24 h later. After 48 h of transfection, 1 μg/mL puromycin (A1113803, ThermoFisher) was used for selection to obtain stable transfection cell lines. Overexpression transfection efficiency was validated by Western blot [47, 48].

Cell groups: Ctrl group (wild-type cells), sgCav-1 group (Cav-1 knockout cells), vehicle group (cells transfected with empty lentivirus), and Cav-1 up group (cells overexpressing Cav-1).

### Oil Red O staining

Cellular lipid accumulation was assessed by Oil Red O staining. 3T3-L1 cells from each group were collected, washed twice with PBS, and fixed with 4% paraformaldehyde (BL539A, biosharp) within 2 h. After three washes with 60% isopropanol (W292907, Sigma-Aldrich), the cells were treated with an Oil Red O staining solution (C0157S, Beyotime) for 15 min. Excess dye was then removed by washing with 70% ethanol. After drying, cells from each group were photographed under an optical microscope (Olympus CK2). Finally, absorbance was measured at 540 nm using a spectrophotometer (Multiskan Spectrum, Thermo Electron Corporation, USA) for quantitative analysis [44].

### Cell proliferation and colony formation experiments

Cell viability was determined using the CCK-8 assay kit (CK04, Dojindo Laboratories). 1 × 10^5^ cells were seeded in a 96-well plate and incubated at 37 °C. Every 24 h, 10 μL of CCK-8 solution and 100 μL of serum-free medium were added. After a 2-h incubation, the optical density (OD) was measured at 450 nm to assess cell viability [49].

For colony formation assays, 1 × 10^5^ cells were seeded in each well of a plate and cultured in medium containing 10% fetal bovine serum for approximately 14 days, with medium changes every 5 days. After 14 days, colonies were fixed with 70% methanol and stained with 0.1% crystal violet (G1064, Solarbio). The colony formation rate was determined by counting the stained colonies [50].

### Lipid and triglyceride assays

To quantitatively analyze the levels of lipids and triglycerides in each cell group, we utilized the Lipid assay kit (ab242305, Abcam) and Triglyceride assay kit (MAK266, Sigma-Aldrich). The experimental protocol followed the instructions provided with the kits [51].

### RT-qPCR

Total RNA was extracted from cell groups using the Trizol kit (T9424, Sigma-Aldrich). The quality and concentration of the RNA were assessed using a UV-visible spectrophotometer (ND-1000, Nanodrop, USA). For mRNA expression analysis, reverse transcription was performed using the PrimeScript™ RT kit (RR014B, TaKaRa, Japan). Real-time quantitative reverse transcription PCR (RT-qPCR) was carried out on the ABI 7500 PCR system (Applied Biosystems, USA) using the TB Green® Premix Ex TaqTM kit (RR420W, TaKaRa, Japan). Relative quantification was achieved by normalizing to the reference gene GAPDH, and the fold change was calculated using the 2^-ΔΔCt^ method, where ΔΔCt = ΔCt experimental group − ΔCt control group, and ΔCt = target gene Ct − reference gene Ct [52]. The primer sequences are provided in Table S2.

### Western blot

Total protein from cells or tissues was extracted using the protein extraction kit (BC3710, Solarbio, China). The samples were centrifuged at 13,000 rpm for 15 min at 4 °C, and the supernatant was collected. Protein concentration was measured using the BCA protein quantification kit (P0010, Beyotime, China). SDS-PAGE was used for protein separation. The separated proteins were transferred onto PVDF membranes (88518, Thermo Scientific). Blocking was performed at room temperature with 5% skim milk (P0216, Beyotime) for 1 h. Subsequently, the membranes were incubated overnight at 4 °C with diluted primary antibodies against Cav-1 (3238, 1:1000, CST), adipose triglyceride lipase (ATGL, 2138, 1:1000, CST), hormone-sensitive lipase (HSL, 4107, 1:1000, CST), FASN (3180, 1:1000, CST), Pparg (orb11291, 1:500, biorbyt), Adiponectin (ab181281, 1:1000, Abcam), FABP4 (ab92501, 1:1000, Abcam), and GAPDH (ab9485, 1:2500, Abcam). After incubation, the membranes were washed three times with TBST (3 × 5 min) and then incubated with the secondary antibody, Anti-Rabbit-IgG (7074, 1/1000, CST), at room temperature for 1 h. Following another three washes with TBST (3 × 5 min), the TBST was discarded, and the PVDF membranes were immersed in an appropriate amount of ECL working solution (WBULS0500, EMD Millipore, USA). The membranes were incubated at room temperature for 1 min, and excess ECL working solution on the PVDF membranes was discarded. The membranes were covered with plastic wrap, placed in a dark box, and exposed to X-ray film for 5–10 min for development and fixation [53]. The Western blot bands were subjected to grayscale quantification using ImageJ analysis software, with GAPDH as the internal control. Please refer to the supplemental materials for full-length western blots.

### Flow cytometry

3T3-L1 cells (1 × 10^5^/well) were collected, centrifuged at 1500 rpm for 5 min, and washed twice with cold PBS. Subsequently, the cells were fixed with 70% ethanol (1012768, Sigma-Aldrich) for 15 min. After another centrifugation at 1500 rpm for 5 min, the cells were washed twice with cold PBS. Then, RNA was removed by incubating the cells with RNaseA (10 μg/μL, EN0531, ThermoFisher) without DNase at 37 °C for 45 min, followed by two washes with cold PBS. After centrifugation at 150 rpm for 5 min, the cells were incubated with NucRed™ Dead 647 ReadyProbes™ reagent (R37113, ThermoFisher) at 4 °C in the dark for 15 min. The percentage of cells in each phase of the cell cycle was quantified using a flow cytometer and analyzed with ModFit LTTM software (Olympus, Japan) [54].

### Immunofluorescence

Slices of Supraspinatus (SS) muscle tissue were prepared. Permeabilization was performed using 0.1% Triton (L885651, Macklin), followed by blocking with 5% BSA for 1 h. 3T3-L1 cells were fixed with 4% paraformaldehyde (BL539A, biosharp) for 15–30 min after being washed with cold PBS. Subsequently, the cells were treated with 0.1% Triton for 15 min. Slices or cells were then incubated overnight at 4 °C with anti-Cav-1 (3238, 1:400, CST) and anti-Perilipin-1 (ab3526, 1:200, Abcam) antibodies. After washing with PBS, the slices or cells were incubated with goat anti-rabbit IgG (H + L) Alexa Fluor® 647 secondary antibody (ab150079, 1/200, Abcam) for 3 h, followed by three washes with PBS. Finally, the slides were mounted using a mounting medium containing DAPI (4083, CST) for fluorescence quenching [55].

For EdU staining, 3T3-L1 cells were seeded in a 24-well plate (2 × 10^5^ cells/well) and incubated for 24 h. The cells were then exposed to 10 μmol/L 5-ethynyl-2’-deoxyuridine (EdU) (A10044, Invitrogen) for 2 h. Nuclear restaining was performed using Hoechst 33342 (62249, Thermo Scientific) [56].

Finally, observations and image acquisition were performed using a laser confocal microscope (LSM 980, ZEISS). Five animals were used for each group, with one slice stained per animal and one field selected for imaging. For cell experiments, three random independent areas were counted. After image acquisition, the number of positive cells was calculated using ImageJ software (National Institutes of Health, USA) [57].

### Animal experimentation and grouping

Male C57BL/6 mice (strain code: 219), aged 6–8 weeks and weighing 22 ± 3 g, were purchased from Beijing Vital River Laboratory Animal Technology Co., Ltd. Initially, the mice in each group were anesthetized by intraperitoneal injection of 0.3% sodium pentobarbital. A longitudinal skin incision was made on the anterior outer side of the left shoulder, followed by a horizontal incision cutting through the deltoid muscle to expose the SS muscle. A 6-0 PDS suture (Z127H, Ethicon) was placed on the SS muscle, and then the SS tendon at the insertional site of the greater tuberosity was cut, and the cartilage layer covering the tendon insertion site was removed using a No.15 blade to expose the underlying cancellous bone. A 1 mm tunnel was made transversely in the humeral head, from back to front, with the entrance of the tunnel located at the site of the SS tendon insertion. The tendon was then passed through the tunnel and reattached to its original insertion site. Finally, the deltoid muscle and skin were sutured layer by layer. All mice were allowed free movement in their cages after surgery. Within the following 3 days, penicillin (P8010, Solarbio) was injected once daily [58].

The experimental groups included: Sham group (sham surgery), Model group (RCI model), Treadmill group (model + treadmill training), antiCav-1 group (model + Cav-1 antibody treatment), and Treadmill + antiCav-1 group (model + treadmill training + Cav-1 antibody treatment).

For the treatment of Cav-1, the Cav-1 antibody (3238, CST) was dissolved and diluted to a final concentration of 2 μM. A weekly intra-articular injection of 5 μL Cav-1 antibody was given to the mice, lasting for either 4 or 8 weeks. In the other groups, an equal volume of PBS was injected into the left shoulder joint. At the end of the experiment, shoulder specimens were collected and stored at −80 °C for subsequent analysis [58].

### Treadmill training

Mechanical load was applied to the mice using an electric treadmill. The treadmill equipment had twelve running lanes, each equipped with an electric shock device at the end. Prior to surgery, mice from both the control and treadmill groups underwent 1 week of adaptive training to familiarize themselves with the treadmill environment. The speed of the treadmill was gradually increased each day, from 5–10 m/min, until all mice were able to adapt to a speed of 10 m/min for 20 min during each session of running.

Starting from the 7th day after surgery, mice in the training group ran on a level (0° inclined) treadmill at a speed of 10 m/min for 20 min each day, 5 days per week. The experiment lasted for a total of 8 weeks. At the end of the experiment, mice from all groups were euthanized by cervical dislocation under anesthesia, and their tissues were collected for subsequent experiments [59].

### Biomechanical testing

For biomechanical testing, each supraspinatus tendon-humerus (STH) complex was carefully dissected under a surgical microscope, removed from the surrounding tissues, and immediately preserved at −80 °C. These specimens were thawed at room temperature in 0.9% saline solution at 37 °C and kept moist throughout the testing period. Prior to testing, all sutures in the STH complex were carefully removed to ensure a clean assessment. The humerus was fixed using sandpaper to secure the supraspinatus tendon. The complex was positioned in a microcomputer-controlled electronic testing system (WD-T, Zhuoji Instruments, Shanghai, China) to perform uniaxial tensile testing at an abduction angle of approximately 60°. The specimens underwent preloading at 0–0.5 N for three cycles before being loaded to failure at a rate of 1 mm/min. The failure load was recorded, and stiffness was calculated as the slope of the best-fit line in the linear region of the force-displacement curve, using software (MATLAB, Natick, USA) according to the method described by Stephen et al. [60].

### Muscle collection and histopathological evaluation

At weeks 4 and 8, bilateral SS muscles of each group of mice were collected and weighed. The wet weight of bilateral SS muscles was calculated as follows: wet weight = ([weight of the right SS muscle − weight of the left SS muscle] / weight of the left SS muscle) × 100%. Tissue histological images of the SS muscles were captured using an Axio Imager 2 microscope (Zeiss, Oberkochen, Germany) and analyzed using ImageJ software. The cross-sectional area (CSA) of muscle fibers was calculated using the formula: CSA = ([right CSA − left CSA] / left CSA) × 100% [61]. Muscle tissue was rapidly frozen and prepared into 10μm-thick frozen sections. Fat infiltration was evaluated using Oil Red O staining. Furthermore, the tissue was fixed in fresh 4% neutral buffered formaldehyde for 24 h, followed by gradient alcohol dehydration, clarification, and routine paraffin embedding. Sections of 5 µm thickness were obtained using a paraffin microtome, baked at 60 °C for 1 h, and then dewaxed with xylene. After hydration, sections were observed using H&E staining (C0105S, Beyotime) and Masson’s trichrome staining (G1340, Solabio).

For H&E staining, the sections were initially immersed in hematoxylin staining solution for 3 min, followed by a 10-s rinse and 10-s differentiation in 1% hydrochloric acid ethanol. After a 1-min distilled water wash, the sections were stained in eosin solution for 1 min, briefly rinsed for 10 s, and then subjected to gradient alcohol dehydration and xylene clarification before being mounted with neutral mounting medium [62, 63].

For Masson’s trichrome staining, the sections were immersed in Bouin’s solution for 15 min, followed by a 5-min rinse with distilled water. The sections were stained with Weigert’s hematoxylin solution for 5 min, rinsed again with distilled water for 5 min, and then immersed in the ponceau-azophloxine solution for 5 min, followed by another 5-min rinse with distilled water. Next, the sections were immersed in phosphotungstic-phosphomolybdic acid and aniline blue dye for 5 min, fixed in 1% acetic acid for 2 min, rinsed briefly for 5 min, and then subjected to gradient alcohol dehydration and xylene clarification before being mounted with neutral mounting medium [18]. The pathological changes were observed under an optical microscope (Olympus CK2).

### Synchrotron radiation micro-computed tomography (SR-μCT)

In weeks 4 and 8 of the experiment, anesthesia was administered to each group of mice, followed by cervical dislocation for euthanasia. Subsequently, specimens of the supraspinatus tendon (SST) insertion site in the SS muscle were scanned. To fix the specimens, they were immersed in 4% neutral buffered formalin for 24 h, and before scanning, they were washed with 0.9% physiological saline to remove residual formalin. The new bone formation in the SST insertion specimens was evaluated using the beamline (BL13W1) of the Chinese Shanghai Synchrotron Facility for X-ray imaging and biomedical applications. The specimens were positioned at the center of a rotation table and scanned within an angular range of 180° with a step size of 0.25°. The beam energy was set at 18.0 keV, the exposure time was 0.5 s, and the sample-to-detector distance was 10.0 cm. A total of 720 X-ray projection images were captured using a charge-coupled device (CCD) detector with a spatial resolution of 3.25 μm per pixel. Dark-field and flat-field images were obtained for the correction of electronic noise and X-ray source brightness variations. The X-ray images of these projections were phase-retrieved using the PITRE software developed by BL13W1 and converted into 8-bit slices. After the noise caused by the grayscale threshold filter was removed, we extracted the bone from the soft tissue. Finally, morphological parameters of the newly formed bone, including bone volume fraction (BV/TV), trabecular thickness (Tb.Th), trabecular number (Tb.N), and trabecular separation (Tb.Sp), were calculated using the data analysis software (CT Analyzer v1.11, Bruker Corporation, Germany) [59].

### Statistical software and data analysis methods

Our study utilized version 4.2.1 of the R language (https://www.r-project.org/), with RStudio (version 2022.12.0-353) as the integrated development environment for R programming. GraphPad Prism 8.0 software was employed for data processing, with quantitative data presented as mean ± standard deviation (Mean ± SD). Non-paired *t*-tests were utilized for comparisons between two groups, while one-way analysis of variance (ANOVA) was employed for comparisons among multiple groups. Levene’s test was conducted to assess the homogeneity of variances. In cases of homogeneity, Dunnett’s T3 and LSD *t*-tests were employed for pairwise comparisons. In cases of heterogeneity, Dunnett’s T3 test was utilized [64]. Chi-squared tests and Spearman correlation were calculated to evaluate the correlation between variables [65]. A significance level of *P* < 0.05 was considered statistically significant for comparisons between the two groups.

## Supplementary information

Supplemental Material(Original WB images)

Supplementary Tables and Figures

## Data Availability

All data can be provided as needed.
