# Supplementary material for: Targeting Caveolin-1 for enhanced rotator cuff repair: findings from single-cell RNA sequencing
Source: Cell Death Discov. 2025 Mar 5;11:88. doi: 10.1038/s41420-025-02359-2 (PMC11882801; doi:10.1038/s41420-025-02359-2)
Supplement: Supplementary file 1 — Supplemental Material(Original WB images) [file 41420_2025_2359_MOESM1_ESM.docx]

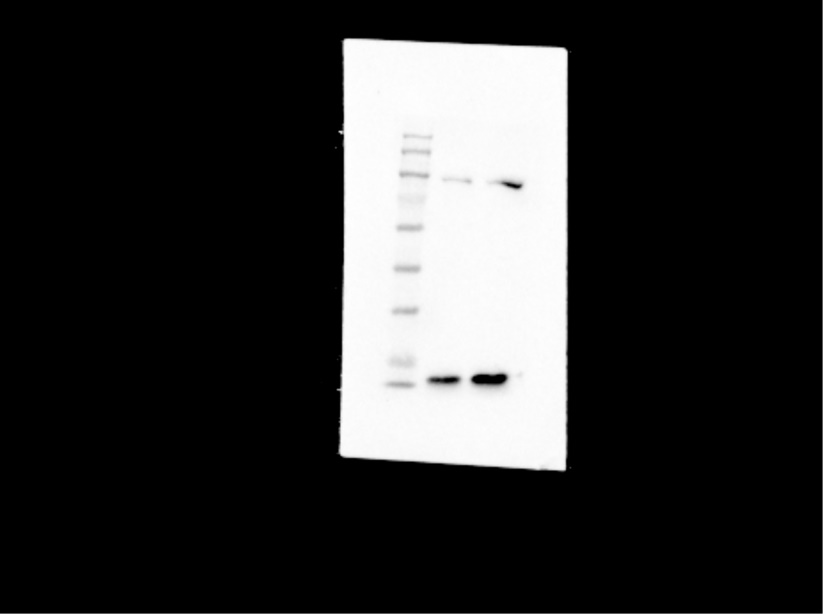


Full length western blots for Figure 1B-1


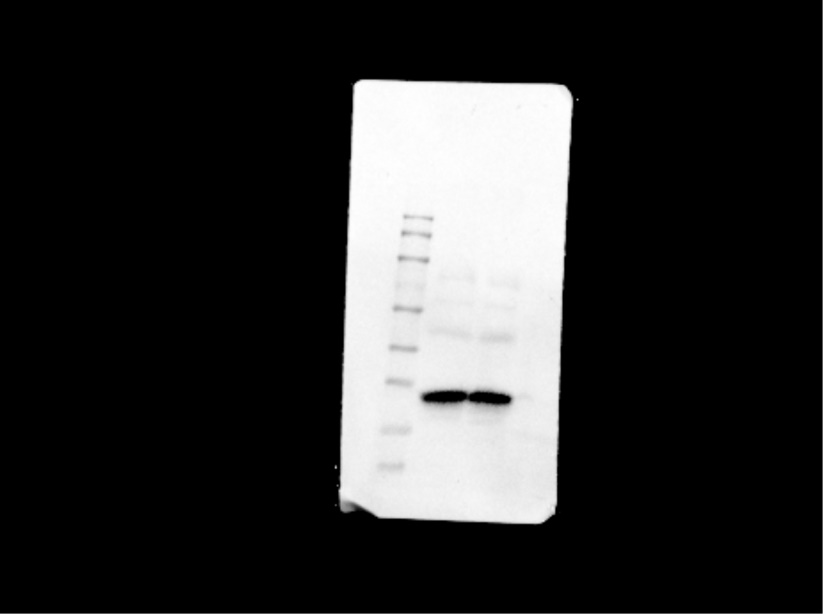


Full length western blots for Figure 1B-2


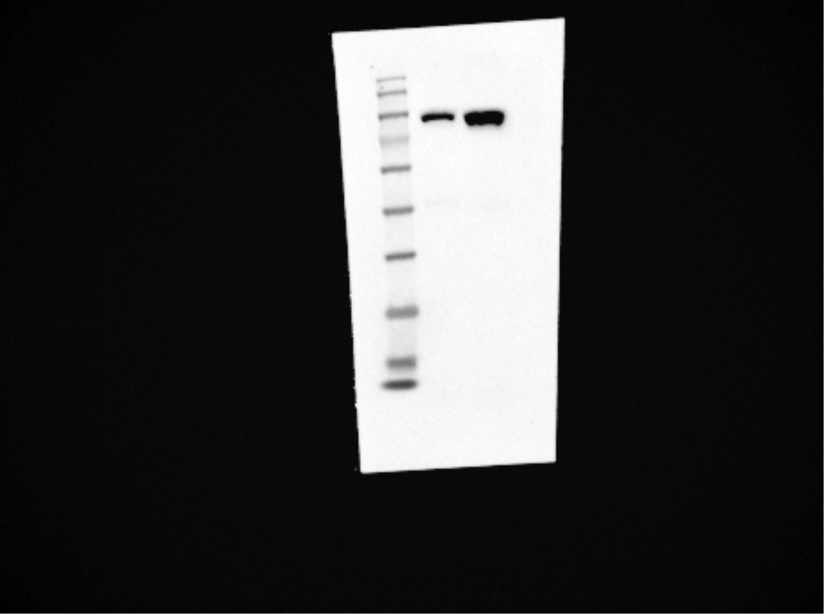


Full length western blots for Figure 1D-1


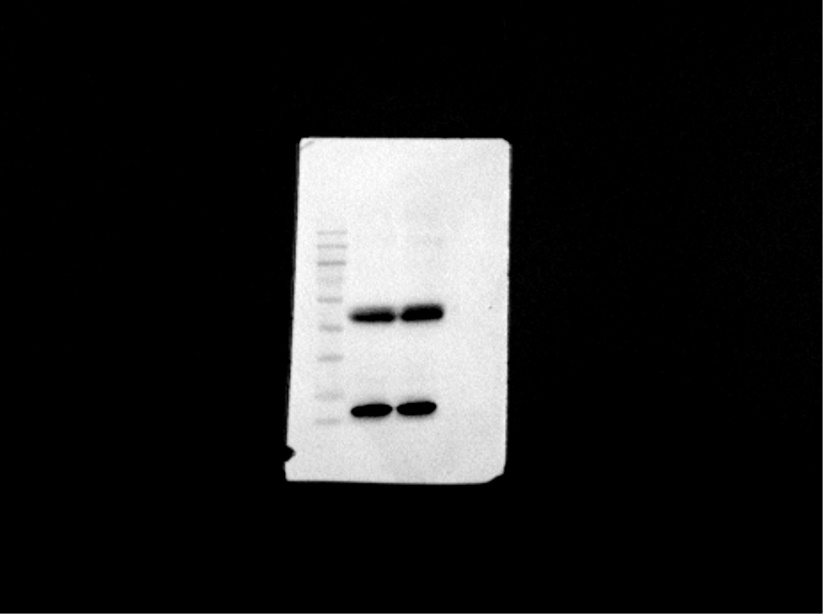


Full length western blots for Figure 1D-2


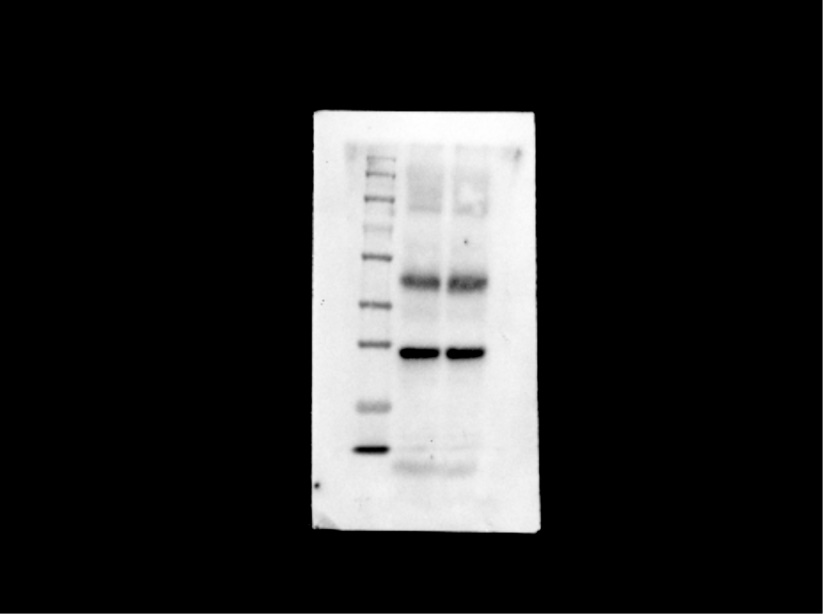


Full length western blots for Figure 1D-3


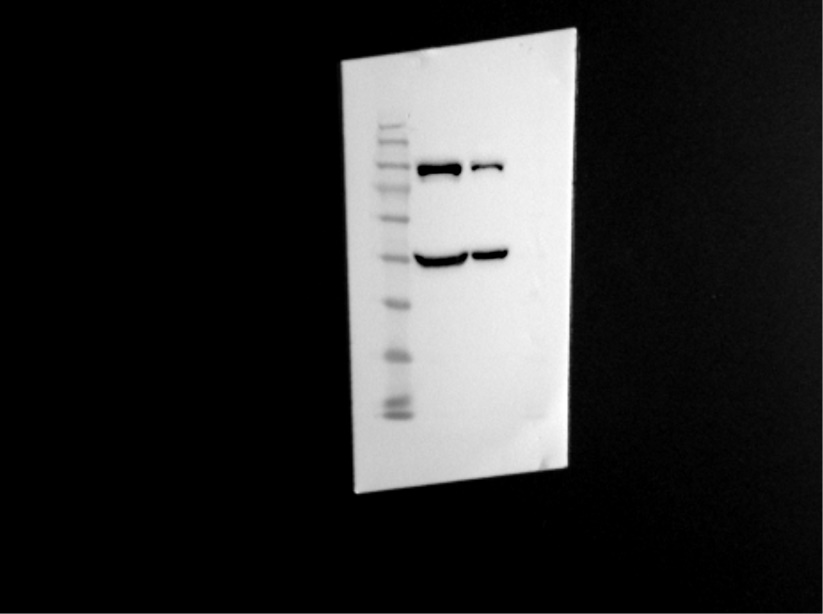


Full length western blots for Figure 5E-1-1


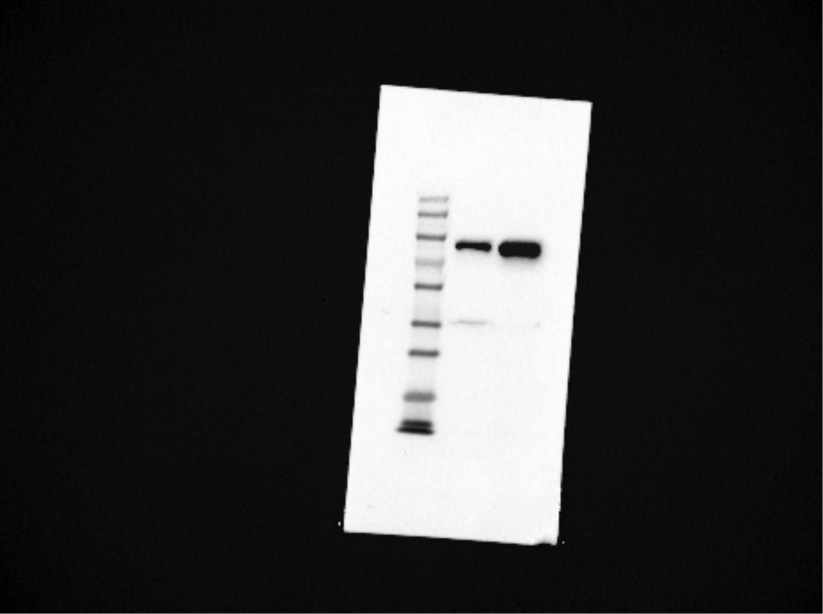


Full length western blots for Figure 5E-1-2


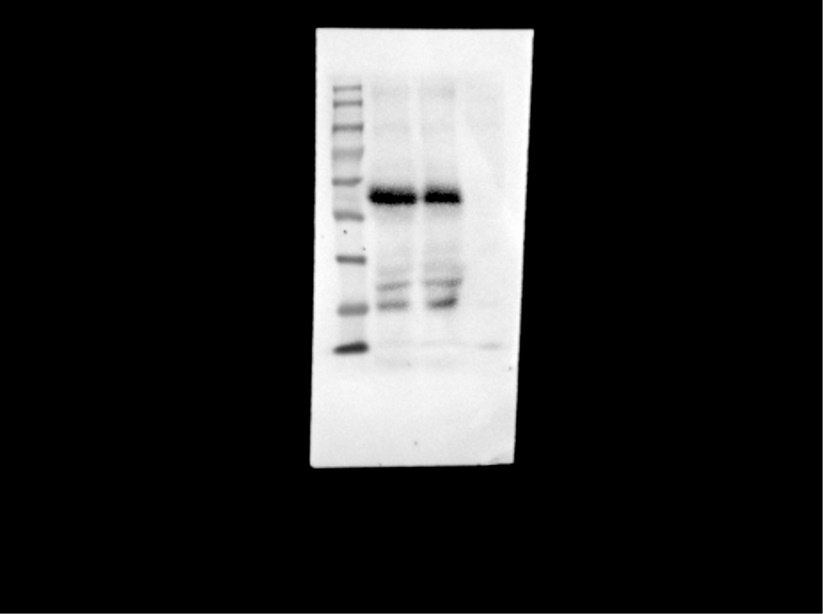


Full length western blots for Figure 5E-2-1


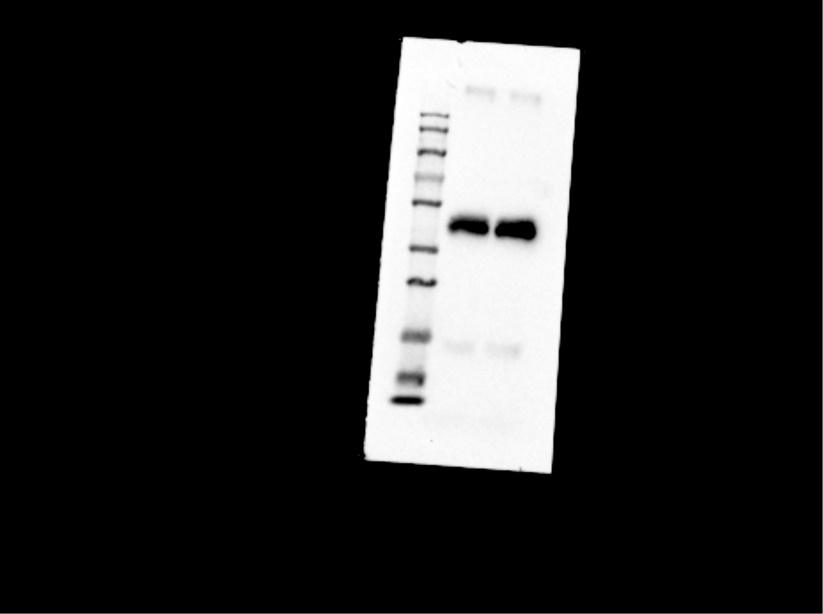


Full length western blots for Figure 5E-2-2


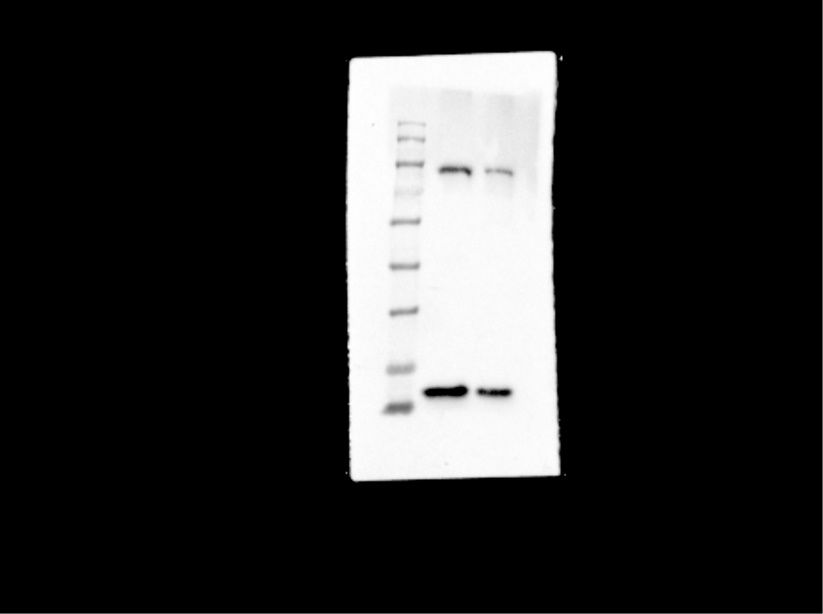


Full length western blots for Figure 5E-3-1


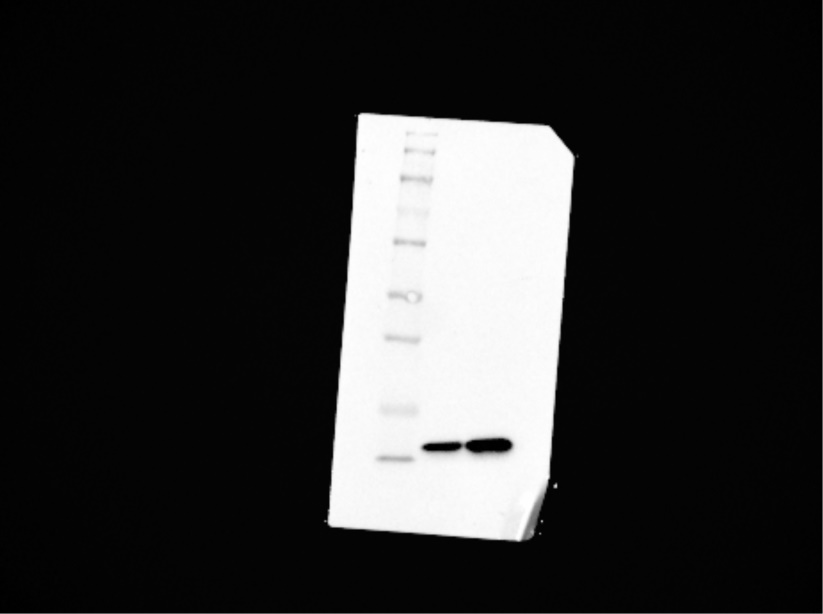


Full length western blots for Figure 5E-3-2


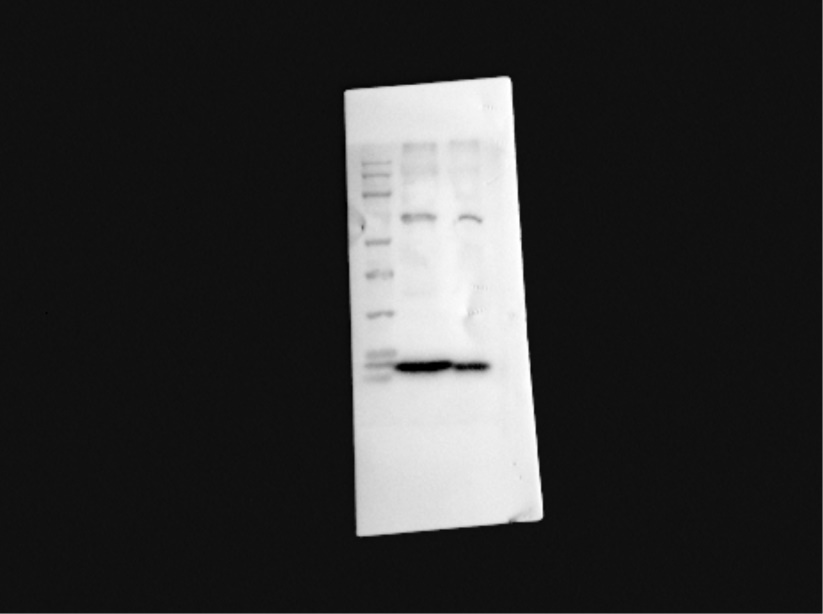


Full length western blots for Figure 5E-4-1


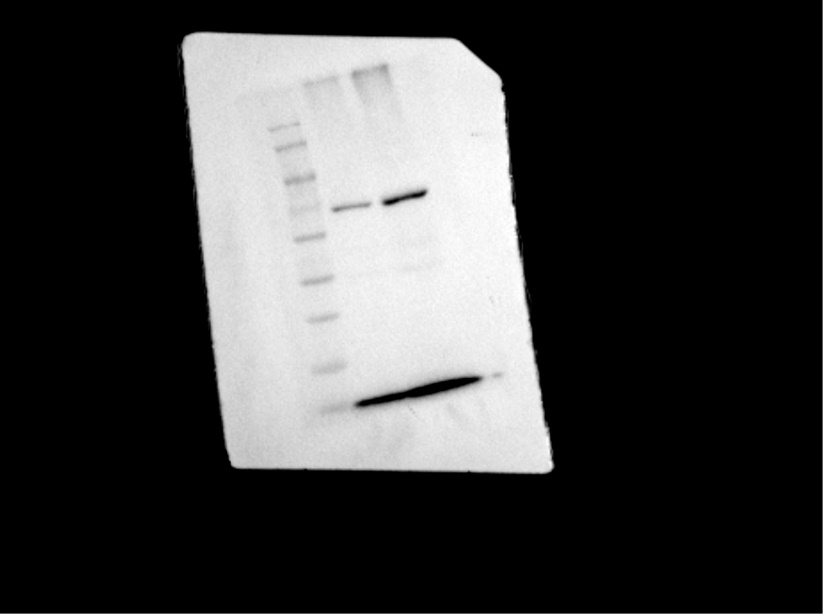


Full length western blots for Figure 5E-4-2


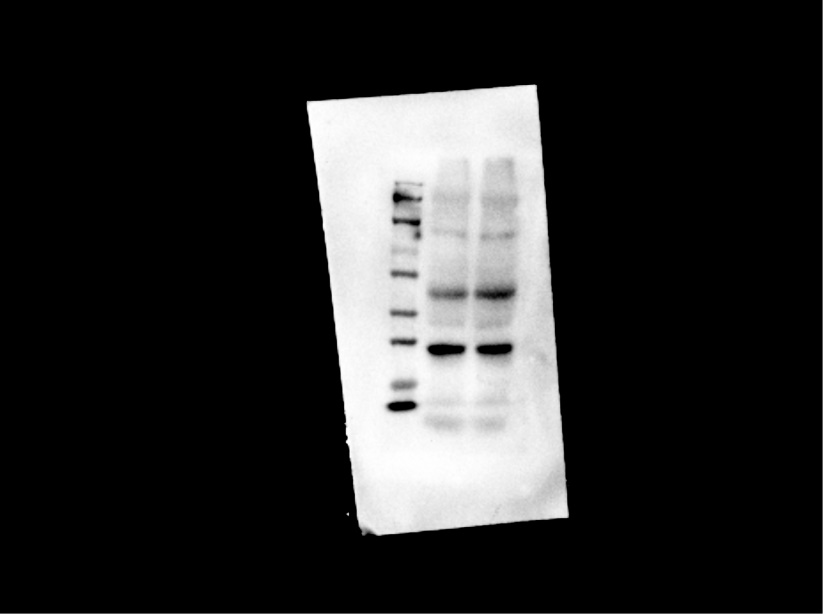


Full length western blots for Figure 5E-5-1


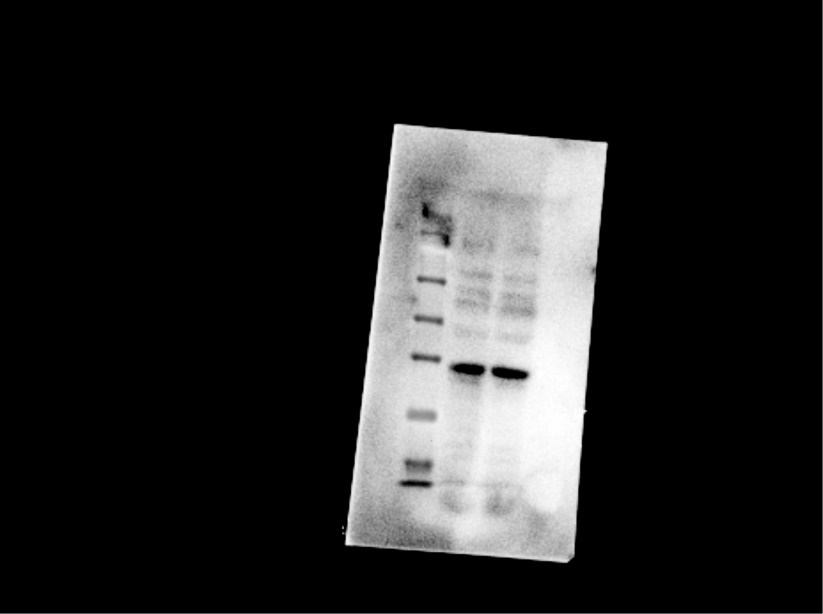


Full length western blots for Figure 5E-5-2


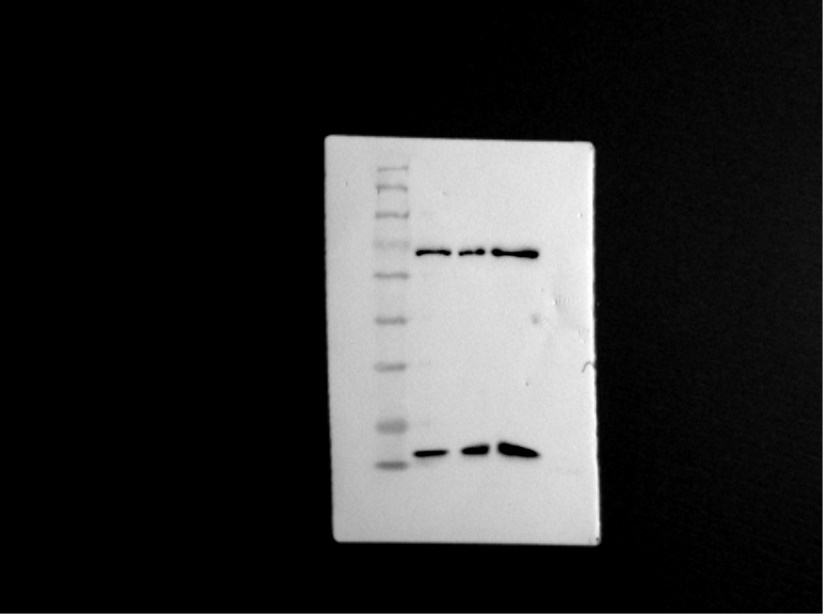


Full length western blots for Figure S2C-1


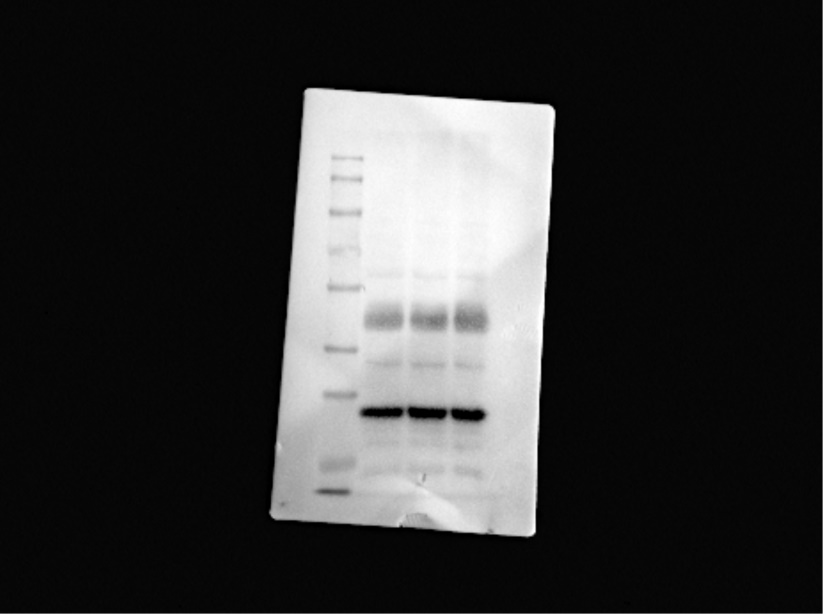


Full length western blots for Figure S2C-2


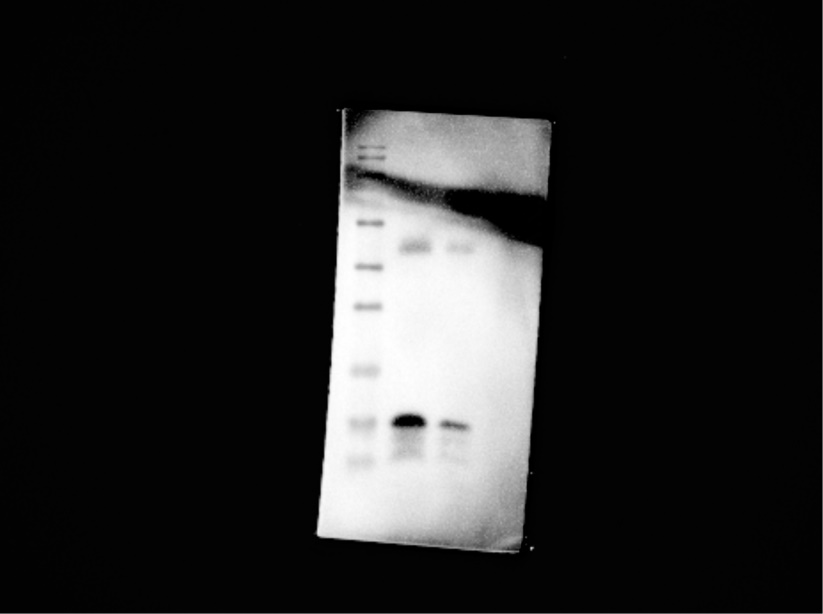


Full length western blots for Figure S2D-1


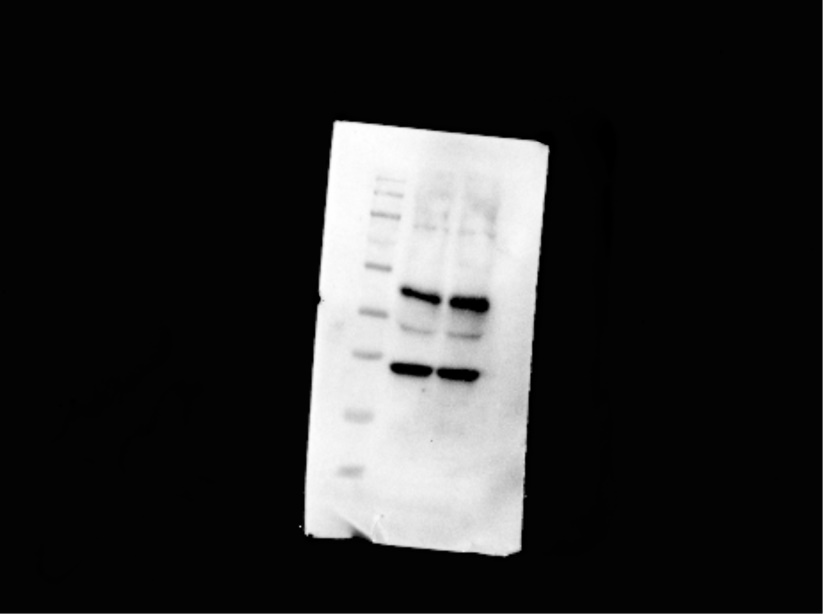


Full length western blots for Figure S2D-2


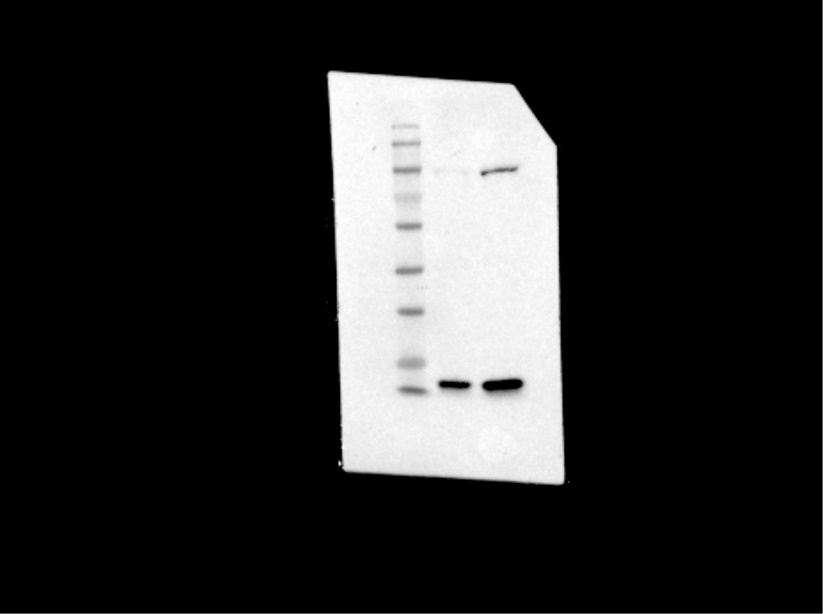


Full length western blots for Figure S2E-1


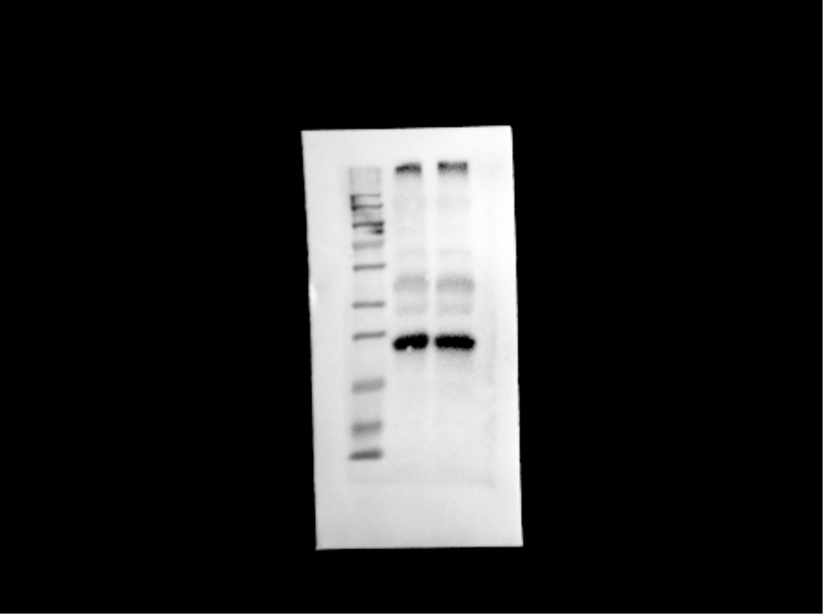


Full length western blots for Figure S2E-2


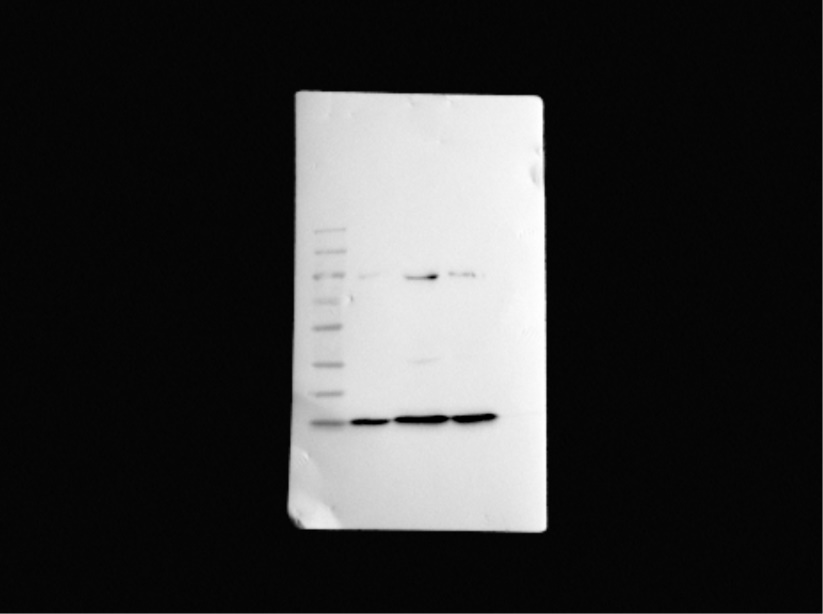


Full length western blots for Figure S4C-1


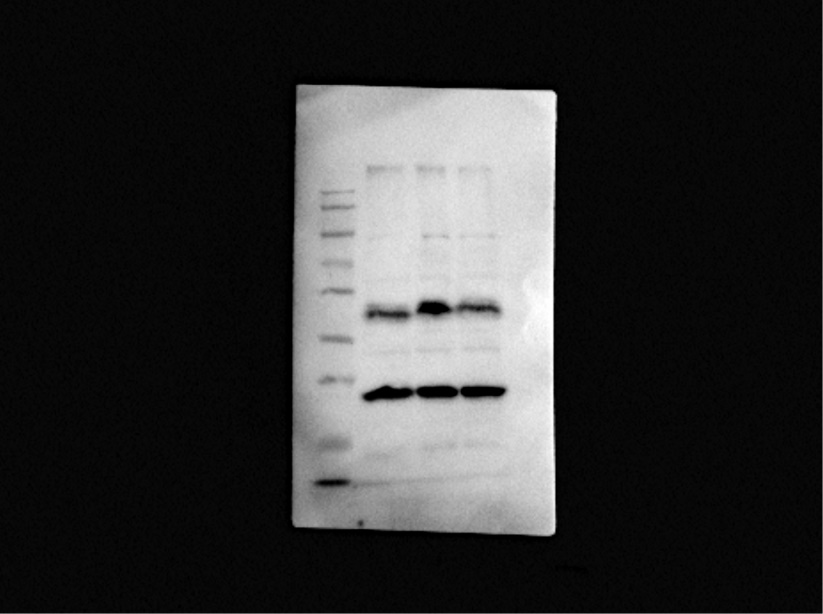


Full length western blots for Figure S4C-2


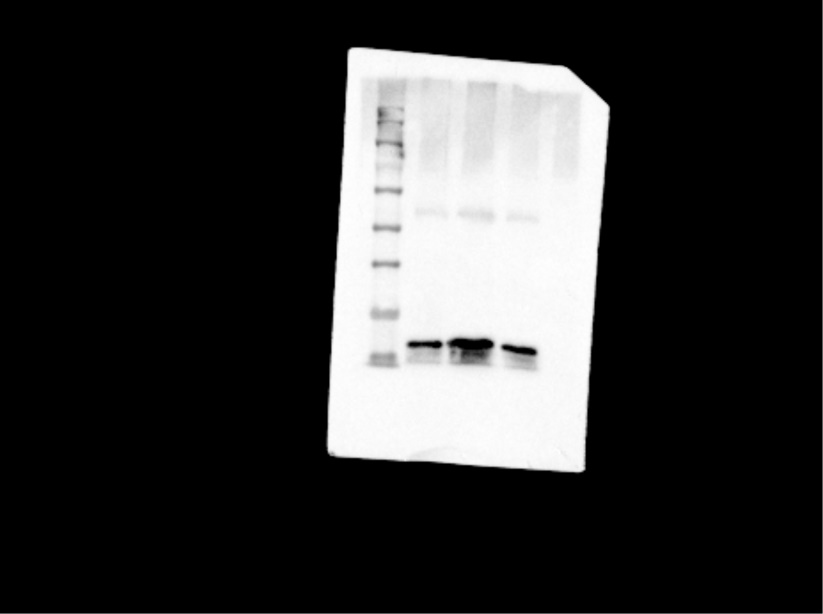


Full length western blots for Figure S4C-3


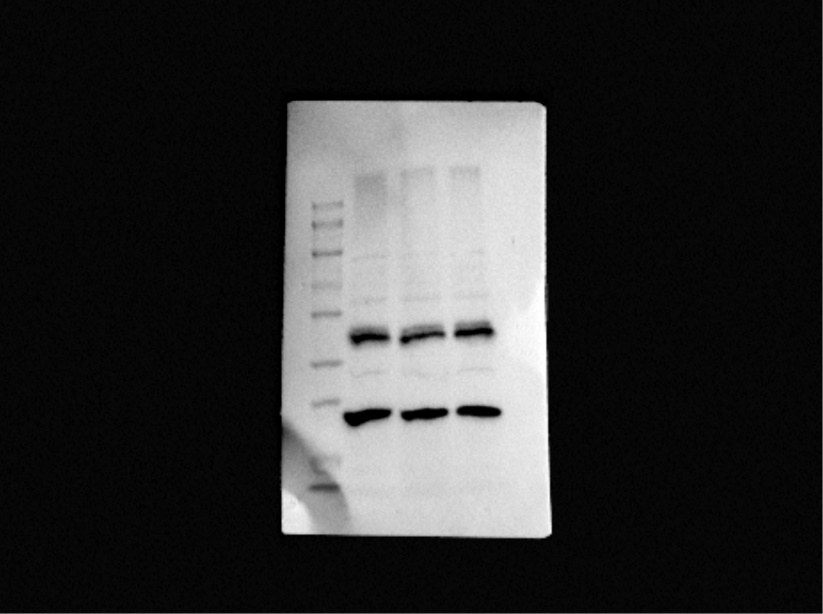


Full length western blots for Figure S4C-4


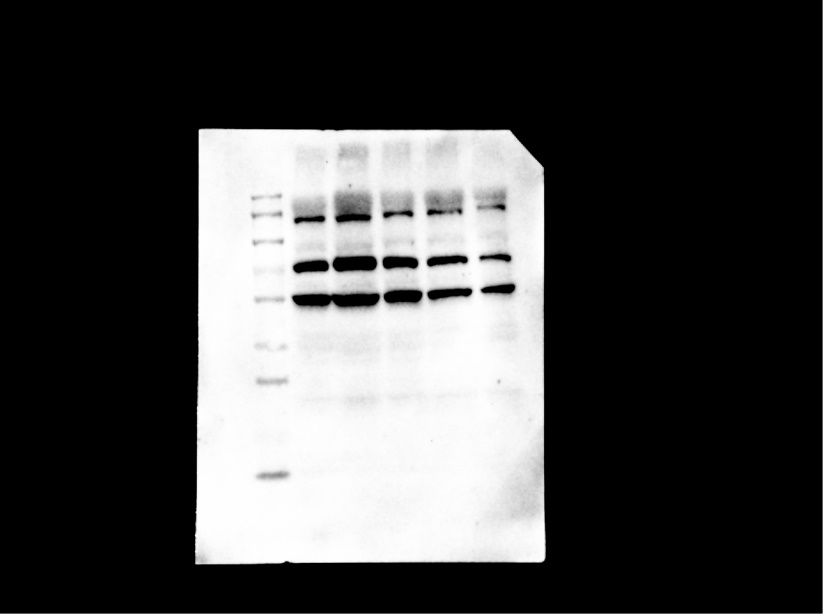


Full length western blots for Figure S5A-1


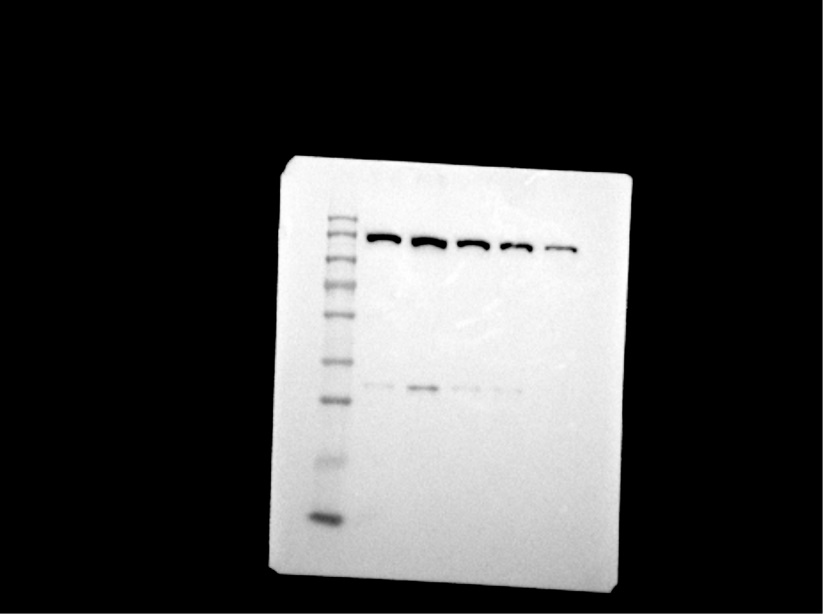


Full length western blots for Figure S5A-2


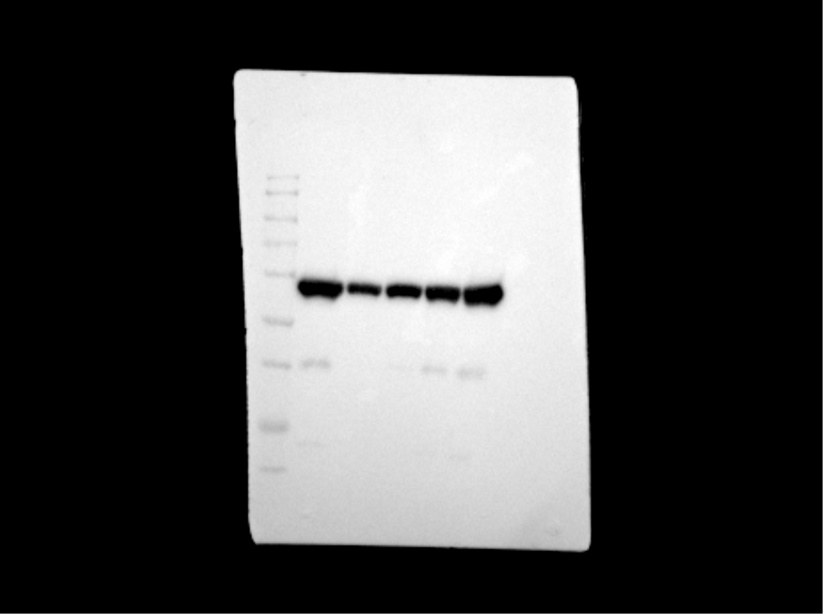


Full length western blots for Figure S5A-3


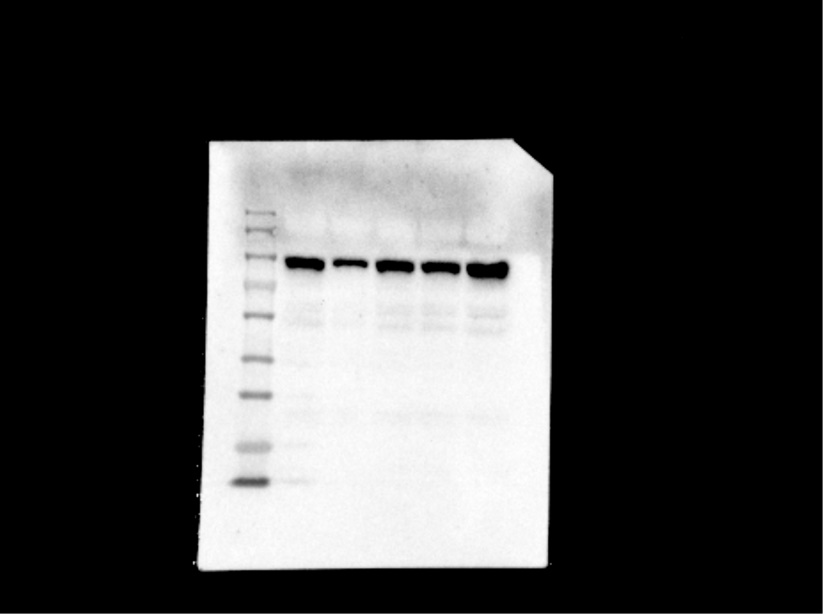


Full length western blots for Figure S5A-4


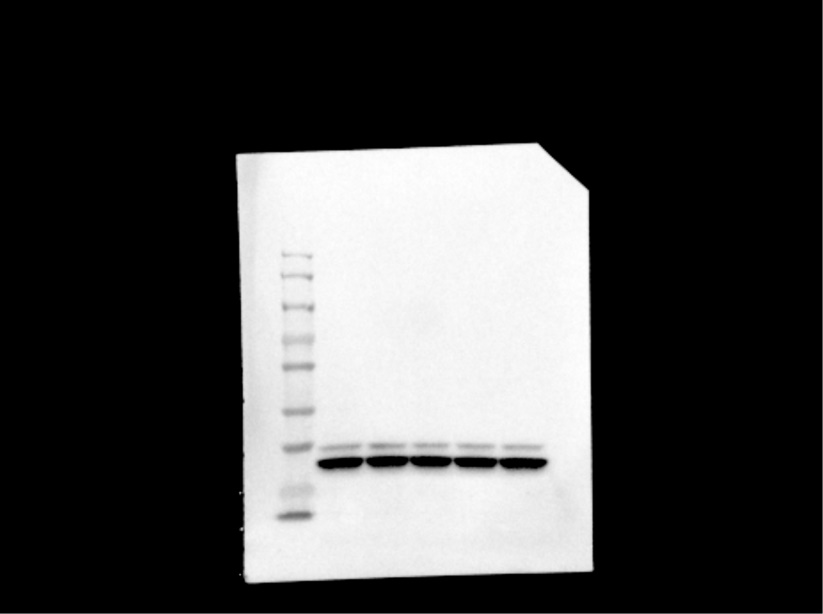


Full length western blots for Figure S5A-5
