# Supplementary material for: Targeting Caveolin-1 for enhanced rotator cuff repair: findings from single-cell RNA sequencing
Source: Cell Death Discov. 2025 Mar 5;11:88. doi: 10.1038/s41420-025-02359-2 (PMC11882801; doi:10.1038/s41420-025-02359-2)
Supplement: Supplementary file 2 — Supplementary Tables and Figures [file 41420_2025_2359_MOESM2_ESM.docx]

**Table S1. Clinical Sample Data**

|  | Sex(cases) | | Age(years) | Disease course (days) | Operative method (cases) | | Degree of injury (cases) | |
| --- | --- | --- | --- | --- | --- | --- | --- | --- |
|  | Male | Female |  |  | Arthroscopy | Small-incision operation | Partial tear | Full-thickness tear |
| Experiment group | 12 | 6 | 37.45 ± 6.13 | 21.71 ± 4.48 | 11 | 7 | 13 | 5 |
| Control group | 10 | 8 | 36.02 ±5.58 | / | / | / | / | / |
| *p* | 0.4941 | | 0.4807 |  |  |  |  |  |

**Table S2. RT-qPCR primer sequences**

| **Gene Name** | **Primer Sequences** |
| --- | --- |
| Caveolin-1(mouse) | Forward: 5'-GCAAAAGTTGTAGCGCCAGG-3' |
|  | Reverse: 5'-ACAGCGGTGGGAAGAAAAGT-3' |
| Caveolin-1(Human) | Forward: 5'-TGTCCGCTTCTGCTATCTGC-3' |
|  | Reverse: 5'-CAGAGGAGTGCTCCGAAGTG-3' |
| GAPDH(mouse) | Forward: 5'-AAGAGGGATGCTGCCCTTAC-3' |
|  | Reverse: 5'-GTTCACACCGACCTTCACCA-3' |
| GAPDH(human) | Forward: 5'-TGCAACCGGGAAGGAAATGA-3' |
|  | Reverse: 5'-GCATCACCCGGAGGAGAAAT-3' |

**
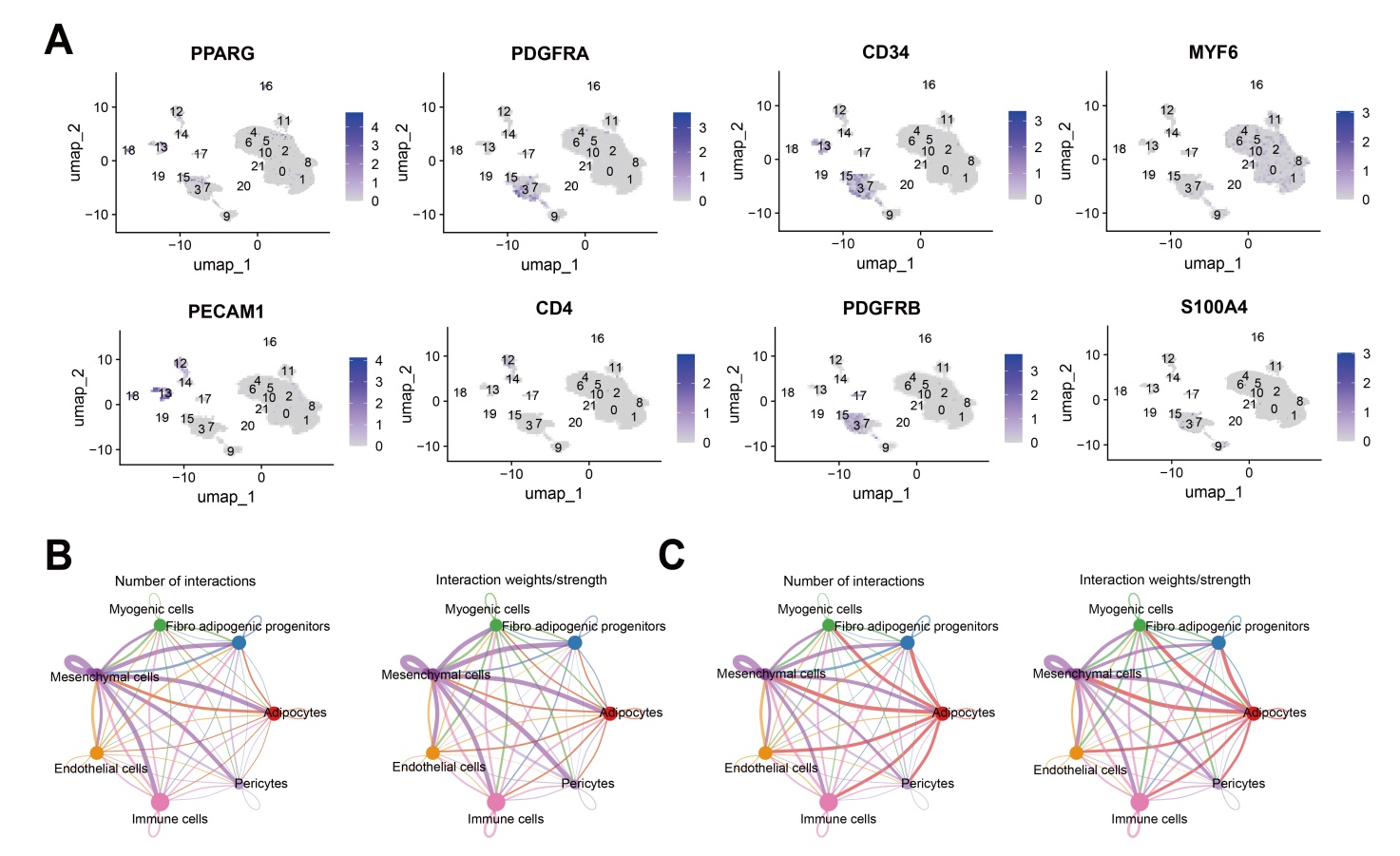
**

**Figure S1. Cell Communication Analysis of scRNA-seq Data**Note: (A) UMAP plots showing the expression levels of different cell marker genes, with varying shades of color representing the intensity of gene expression; (B-C) Cell communication analysis in samples from the Control group (B) and the RCI group (C), where the thickness of the lines represents the quantity or strength of interactions, and the colors indicate different cell types.

**
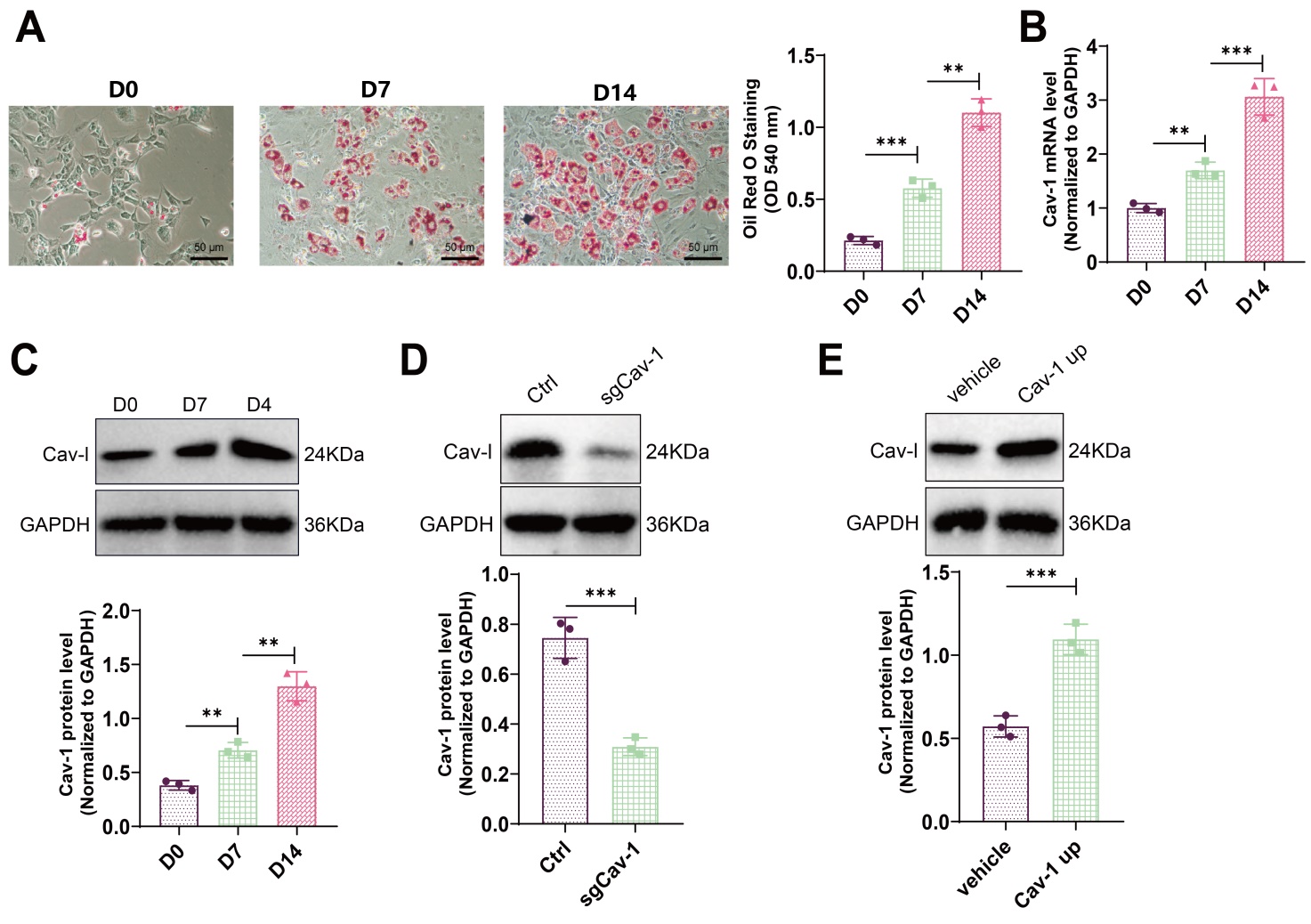
**

**Figure S2. Association of Cav-1 with lipid accumulation in 3T3-L1 cells.**

Note: (A) Oil Red O staining to assess the differentiation capacity of 3T3-L1 cells, Scale bar = 200 μm; (B) RT-qPCR to examine the expression of Cav-1 mRNA during 3T3-L1 cell differentiation; (C) Western blot analysis of Cav-1 expression during 3T3-L1 cell differentiation; (D) CRISPR-Cas9-mediated knockout of Cav-1 expression, with Western blot analysis to determine the efficiency of Cav-1 knockout in 3T3-L1 cells; (E) Overexpression of Cav-1 in 3T3-L1 cells via lentiviral transduction, with Western blot analysis to verify Cav-1 transfection efficiency. *** indicates *P* < 0.001, ** indicates *P* < 0.01. Cell experiments were performed in triplicate.

**
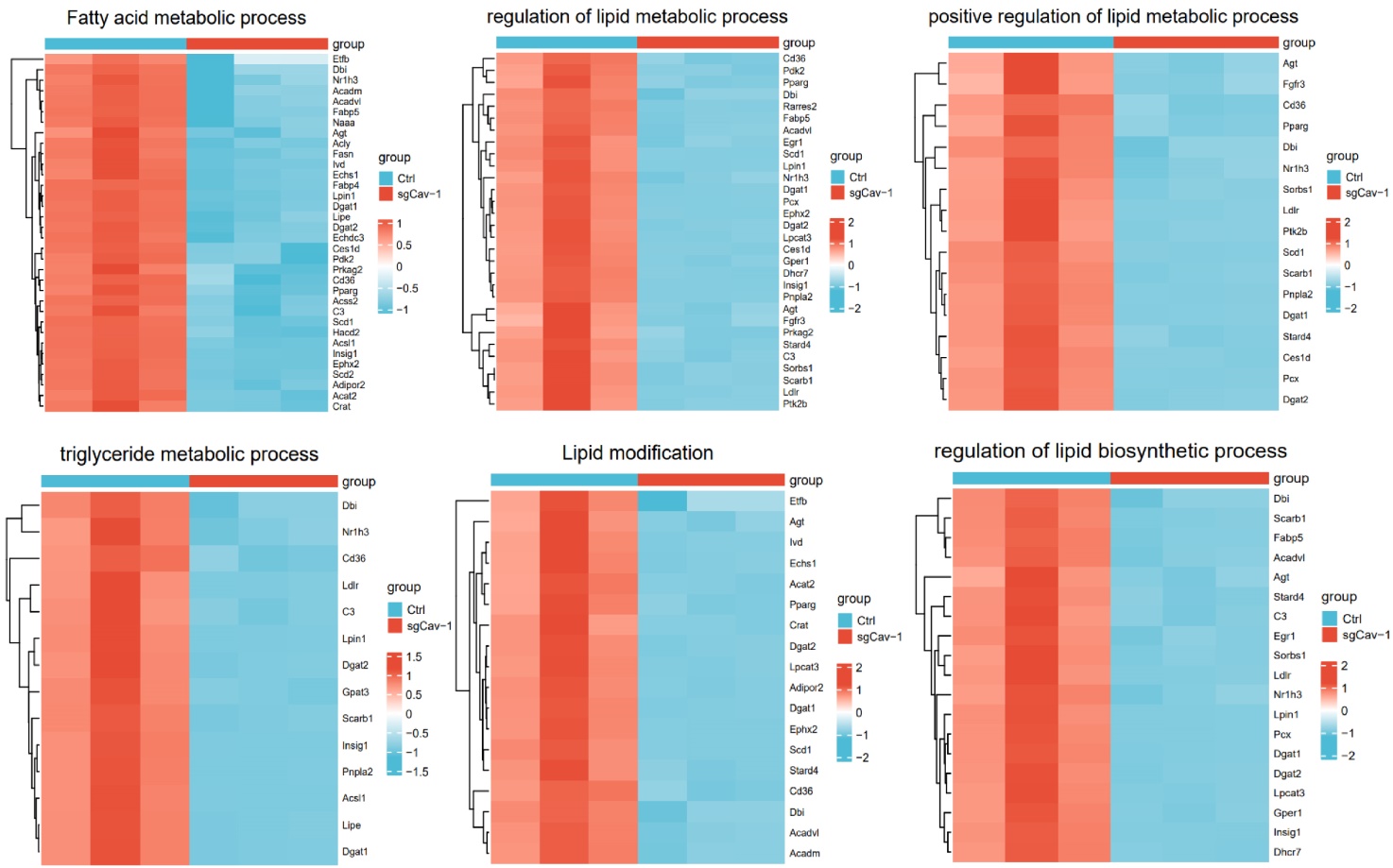
**

**Figure S3. Expression of lipid metabolism-related genes after Cav-1 knockout.**

Note: Transcriptome sequencing analysis revealed the expression patterns of genes related to Fatty acid metabolic process, alcohol metabolic process, positive regulation of lipid metabolic process, triglyceride metabolic process, Lipid modification, regulation of lipid biosynthetic process, etc., after Cav-1 knockout.

**
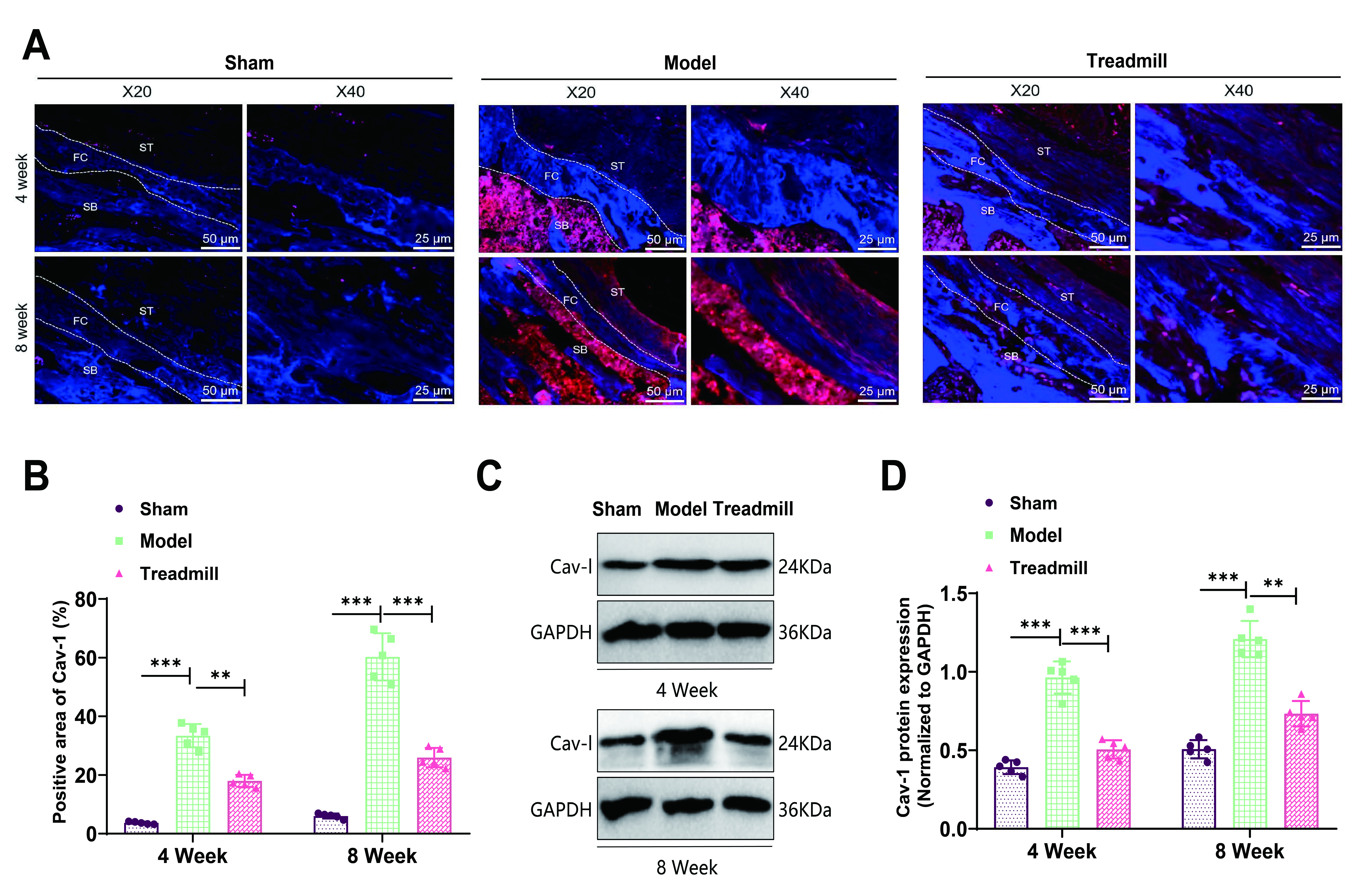
**

**Figure S4. Regulation of Cav-1 expression in the RC site by exercise.**

Note: (A-B) Immunofluorescence staining detected the expression of Cav-1 in the RC injury sites in each group at the 4th and 8th weeks after surgery; (C-D) Western blot analysis of the protein expression levels of Cav-1 in the RC injury sites at the 4th and 8th weeks after surgery in each group. *** indicates *P* < 0.001, ** indicates *P* < 0.01, and n = 5 in animal experiments.

**
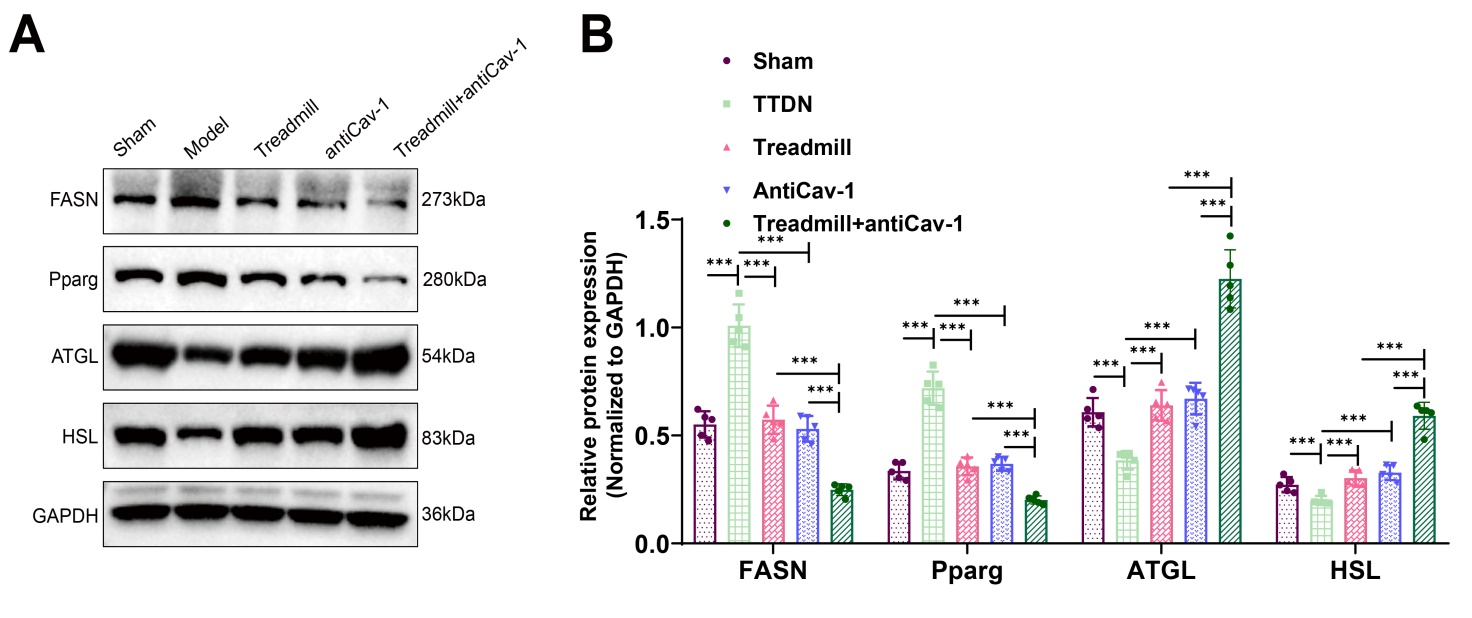
**

**Figure S5. Cav-1 Regulates lipid accumulation and adipogenesis in muscle fat cells.**

Note: (A-B) Expression levels of FASN, Pparg, ATGL, and HSL in muscles of different groups of mice were detected by Western blot. *** indicates *P* < 0.001, and n = 5 in animal experiments.
